# A Quantitative Systems Pharmacology Model of T Cell Engager Applied to Solid Tumor

**DOI:** 10.1208/s12248-020-00450-3

**Published:** 2020-06-12

**Authors:** Huilin Ma, Hanwen Wang, Richard J. Sove, Mohammad Jafarnejad, Chia-Hung Tsai, Jun Wang, Craig Giragossian, Aleksander S. Popel

**Affiliations:** 1 Department of Biomedical Engineering, Johns Hopkins University School of Medicine, Baltimore, Maryland USA; 2 Biotherapeutics Discovery Research, Boehringer Ingelheim Pharmaceuticals, Inc., Ridgefield, Connecticut USA; 3 Department of Oncology and Sidney Kimmel Comprehensive Cancer Center, Johns Hopkins University, Baltimore, Maryland USA

**Keywords:** cancer systems biology, immuno-oncology, cancer immunotherapy, computational biology, colorectal cancer

## Abstract

Cancer immunotherapy has recently drawn remarkable attention as promising results in the clinic have shown its ability to improve the overall survival, and T cells are considered to be one of the primary effectors for cancer immunotherapy. Enhanced and restored T cell tumoricidal activity has shown great potential for killing cancer cells. Bispecific T cell engagers (TCEs) are a growing class of molecules that are designed to bind two different antigens on the surface of T cells and cancer cells to bring them in close proximity and selectively activate effector T cells to kill target cancer cells. New T cell engagers are being investigated for the treatment of solid tumors. The activity of newly developed T cell engagers showed a strong correlation with tumor target antigen expression. However, the correlation between tumor-associated antigen expression and overall response of cancer patients is poorly understood. In this study, we used a well-calibrated quantitative systems pharmacology (QSP) model extended to bispecific T cell engagers to explore their efficacy and identify potential biomarkers. In principle, patient-specific response can be predicted through this model according to each patient’s individual characteristics. This extended QSP model has been calibrated with available experimental data and provides predictions of patients’ response to TCE treatment.

**Electronic supplementary material:**

The online version of this article (10.1208/s12248-020-00450-3) contains supplementary material, which is available to authorized users.

## Introduction

Colorectal cancer (CRC) is the third most common cancer diagnosed in the USA for both men and women. Based on the American Cancer Society’s estimates, there were about 100,000 new cases of colon cancer and 50,000 new cases of rectal cancer in 2019. Colorectal cancer is the third leading cause of cancer-related deaths for men and women and the second most common cause of cancer deaths when genders are combined. However, due to advances in screening techniques and improvements in treatments over the last decades, the overall death rate from colorectal cancer has been falling (1). Chemotherapy and targeted therapy have shown great potential to improve overall survival, but the side effects and the development of tumor resistance constrain their applications and development prospects (2). Recently, several immune checkpoint inhibitors such as anti-PD-1, anti-PD-L1, and anti-CTLA4 monoclonal antibodies (mAbs) have shown high clinical efficacy particularly in melanoma and in lung cancer (3–5). However, existing immunological checkpoint therapy has little effect on colorectal cancer, especially for metastatic CRC (mCRC) patients with proficient mismatch repair (pMMR) or microsatellite stable (MSS) tumors. More effective therapeutic strategies are urgently needed for these patients (6,7). All abbreviations are described in Table S1.

T cell bispecific antibodies, known as bispecific T cell engagers (TCEs), are a class of engineered bispecific monoclonal antibodies, which can simultaneously bind to a tumor-selective cell surface antigen and receptor (TCR)–associated protein CD3 to activate T cells in an MHC I–independent pathway. The following release of cytokines, cytotoxic granules, and chemokines and proliferation of cytotoxic T cells lead to cancer cell apoptosis. Catumaxomab, the first approved bispecific antibody, targets epithelial cell adhesion molecule (EpCAM) in cancer cells, and CD3 in T cells showed promising therapeutic effects in the treatment of malignant ascites. However, the major side effect of catumaxomab was from the off-target binding of its active Fc region to FcγR-expressing Kupffer cells in the liver, leading to severe T cell–mediated hepatotoxicity. In addition, catumaxomab was withdrawn in 2017 for commercial reasons, but the outstanding therapeutic effect of catumaxomab continues to inspire the development of bispecific antibodies. Blinatumomab, an anti-CD19 and anti-CD3 bispecific antibody, has been approved by FDA for the treatment of B cell acute lymphocytic leukemia. This antibody is made up of two single-chain variable fragments (scFv) connected by a short peptide linker without an IgG Fc domain. This design reduces the risk of developing detrimental downstream immune responses caused by other cells that are activated by Fc domain; however, this also reduces the serum half-life of blinatumomab to about 2 h in humans. A continuous intravenous infusion device is needed for administrating blinatumomab, which may limit its broader application (8). In addition, TCEs have shown less success in solid tumors than hematologic malignancies, most likely due to the poor T cell infiltration (2).

Despite these challenges, several TCEs have entered clinical trials and there are many more ongoing studies (8). There are now over 20 technology platforms for creating and designing TCEs (9). For colorectal cancer, Bacac *et al.* and Lehmann *et al.* have reported the development of a novel T cell bispecific CEA-TCB (T cell bispecific) antibody (cibisatamab, RG7802, RO6958688) for targeting carcinoembryonic antigen (CEA) on tumor cells and CD3 on T cells (10,11). The activity of their CEA-TCB was assessed using 110 colorectal cancer cell lines. High potency was demonstrated in cell lines with high CEA expression (> 10,000 CEA-binding sites/cell). Results showed promising antitumor activity of TCEs against CRC both *in vitro* and *in vivo*. Herrmann *et al.* reported the ability of MT110, an epithelial cell adhesion molecule (EpCAM)/CD3-a antibody, to eliminate colorectal tumor initiating cells (12). The activity of MT110 is strongly dependent on EpCAM expression, and the most frequent EpCAM expression in colorectal cancers makes it a good candidate for this treatment.

Despite the recent progress in TCE development, there is a lack of good predictive biomarkers that can efficiently distinguish responders from non-responders (13). Many new colorectal biomarkers for earlier diagnosis, selection of therapy, and prognosis of colorectal cancer have been identified by recent advances in the molecular subtypes of colorectal cancer, such as methylation of DNA and micro-RNA biogenesis. However, these biomarkers only showed promising results in small-scale studies. Large-scale studies are indispensable for validating their effectiveness. This is an area where employing quantitative systems pharmacology (QSP) models could be constructive and lead to further progress.

Previous studies have demonstrated QSP modeling as a promising approach for addressing current challenges in translational pharmacology (14–20). A mechanistic PK/PD model was used by Betts *et al.* to characterize the *in vivo* PK/PD relationship for a P-cadherin/CD3 bispecific construct in mouse (21). Yuraszeck *et al.* successfully used their QSP model to identify key drivers of response to blinatumomab (22). Demin *et al.* also reported using a QSP model to demonstrate that treatment outcome of blinatumomab is dependent on target expression, level of immune cells, disease progression rate, and expression of PD-L1 on leukemic cells (23). However, these studies focused on either the efficacy in mice or hematological malignancy. A human QSP model to simulate TCE treatment for solid tumors is currently lacking. Our recent study has demonstrated the development of a QSP model to explore the anti-tumor immune response in human non-small cell lung cancer (NSCLC) (24). The model has been calibrated with the available clinical data. Potential biomarkers as well as patient-specific response based on the patient parameters were identified successfully by this model. The model thus provides a solid starting point for modeling tumor immunity and response to immunotherapy to identify biomarkers for different cancer types and perform virtual clinical trials to predict the response in a large cohort of virtual patients.

In this work, we have extended our QSP model by adding a module describing TCE immunotherapy and applied it to colorectal cancer in human. As an important feature of TCEs, the activation of both effector T cells (Teffs) and regulatory T cells (Tregs) is included in this model (25). Taken together, this extended model aims to provide understanding of the complex processes and identify important biomarkers associated with the outcomes of TCE treatment. The validation of these identified biomarkers is essential for novel drug design and for design and analysis of clinical trials.

## Method

### Model Structure

**Fig. 1:**
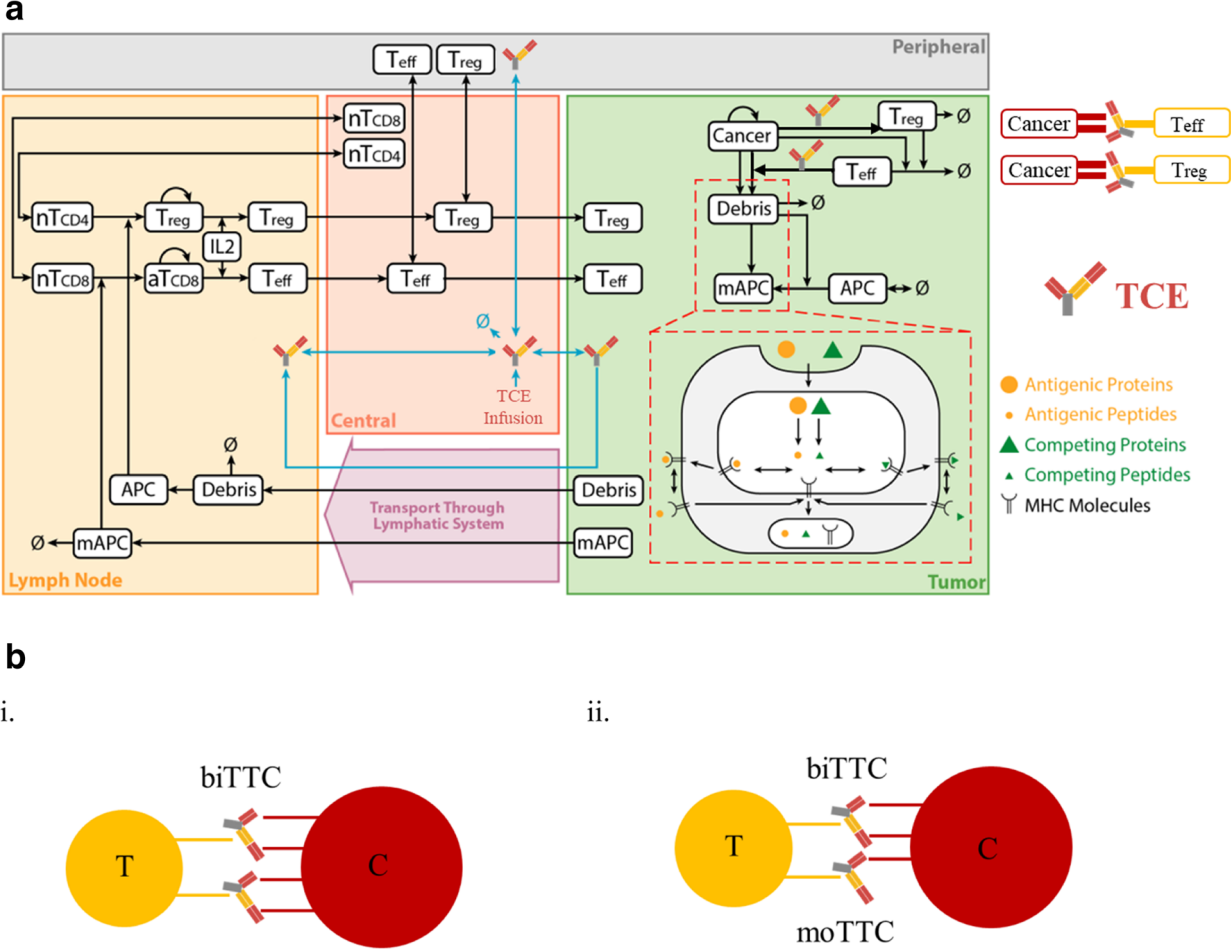
**a** Diagram of the main cellular and molecular interactions implemented in the model. The figure is adapted from reference (24). **b** (i) Structure of TCE ternary complex type 1—bivalent binding (biTTC). (ii) Structure of TCE ternary complex type 2—monovalent binding (moTTC)

The quantitative systems pharmacology model was developed by Jafarnejad *et al.* to study the anti-PD-1 therapy in the context of NSCLC, and detailed governing equations have been formulated and explained in detail (24). Four compartments are included in this model as central (blood), peripheral (other tissues and organs), tumor, and tumor-draining lymph node (TDLN) to represent the patient, and the whole model was defined by a system of ordinary differential equations (ODEs) and algebraic equations. The model has a modular structure to make it easier to add additional modules or modify existing ones, and it includes cancer cell, T cell, immune checkpoint, antibody PK, and antigen presentation modules. Each module represents the dynamics of one major cellular and molecular species such as T cells, cancer cells, antigen-presenting cells, antigens, checkpoint ligands and receptors, and antibodies. This cellular- and molecularly detailed model makes it easier to expand with additional modules describing other species or therapeutic agents. To incorporate TCEs, a new TCE-centered module describing its dynamics was added, and the pharmacokinetics was added to previously published antibody PK module (Fig. 1a). With the inclusion of TCE, the model comprises 68 ODEs and 105 algebraic equations in total. Full equations describing reactions and model parameters were reported by Jafarnejad *et al.* (24); however, for the sake of completeness and for this paper to be self-contained, in the Supplementary Information, we present the Systems Biology Markup Language (SBML) code for the entire model, including all newly added equations for TCE. The SBML version of the computer code includes all equations, rules, and events of tumor growth process, antigen processing and presentation, T cell activation, proliferation, and distribution. In this study, we focused on cibisatamab, RO6958688; RG7802, which is bivalent for the target carcinoembryonic antigen (CEA) on cancer cells and monovalent for CD3 on T cells. PK parameters were fitted to the data reported and the simulated plasma concentration of cibisatamab together with the clinical measurements at dose levels of 80, 160, 200, 300, 400 mg in the central compartment (*Vc* = 3.45 L) (Fig. S1). In our model, we considered the CD3 expression on both Teff and Treg cells; binding to CD3 on Teff or Treg leads to distinct events and will be discussed in detail. However, it should be noted that the model is general and could be readily adapted to apply to other TCEs.

### TCE-Induced Ternary Complex Formation in Tumor Compartment

A TCE module was developed to describe the binding of TCEs to CEA on cancer cells and CD3 on T cells in the tumor compartment to form bivalent TCE ternary complex (biTTC). The formation of stable biTTCs was assumed to drive cancer cell killing by translating the density of biTTCs to the TCE efficacy using the Hill equation. Description of all parameters and abbreviations is presented in Tables S1 and S2. In the tumor compartment, TCEs can bind to CEA or CD3, respectively, to form CEA-TCE or CD3-TCE dimers or bind to CEA and CD3 simultaneously to form the functional biTTCs or nonfunctional monovalent TCE ternary complex (moTTC) (Fig. 1b). Binding of TCEs to CD3 on Teff or Treg cells was determined by kon_CD3TCE, koff_CD3TCE, and number of CD3 on the surface of Teff or Treg cells. Similarly, the other two arms of TCE can bind to one or two CEA on the surface of cancer cells depending on the kon_CEATCE, koff_CEATCE, and intrinsic antibody cross-arm binding efficiency *λ*, which has been explored by Harms *et al.* (26). The expressions of CD3 and CEA on cell surface were taken from literature (10,27–30). The expression can vary widely for different patients and thus was added to the list of parameters for sensitivity analysis.

### TCE Module Calibration

The TCE module was first used to explore the effect of TCE dosing and CEA expression on the Hill function used to translate biTTC formation to TCE efficacy. For this purpose, the TCE module is composed of the tumor compartment only. This mini-model was calibrated based on the results of Bacac *et al.* and Lehmann *et al.* (10,11) to identify several important parameters that determine the efficacy of TCEs such as the Hill equation parameters of biTTC density for half-maximal T cell killing, intrinsic antibody cross-arm binding efficiency *λ*, Hill coefficient, and enhanced cancer cell killing rate induced by TCEs. Some of these parameters are part of the parameter sensitivity analysis. Specified amounts of CD8+ T cells, peripheral blood mononuclear cells (PBMCs), and cancer cells treated with different TCE concentrations in a fixed effector vs target cells (E/T) ratio proposed by Bacac *et al.* and Lehmann *et al.* were added to this module. The mini-model was then fitted to *in vitro* experimental data (10).

### Parameter Sensitivity Analysis

Parameter sensitivity analysis (PSA) was performed to assess the sensitivity of the QSP model with incorporated TCE module to a set of parameters such as cancer cells killing rate by effector T cells, tumor growth rate, antigen–MHC II binding affinity, tumor mutational burden (TMB), antigen expression level on cancer cells, CD3 expression level on Teff cells and Treg cells, CEA-TCE, and CD3-TCE binding affinity, as some of these parameters may vary widely among different patients and result in significantly different therapeutic effects. Latin hypercube sampling (LHS) was used to assign the values for this set of parameters with uniform transformation to study the effect of these inputs on the model outcomes such as tumor volume, Teff/Treg cell ratio in tumor compartment, and CD8+ T cell clonality in blood. Partial rank correlation coefficient (PRCC) analysis was performed to identify the most influential factors from the simulation results and was implemented by using MATLAB (MathWorks, Natick, MA) Global Optimization Toolbox.

### Statistical Analysis

Statistical analysis was performed for virtual patients’ subcohorts. Virtual patients were divided into responders including complete response/partial response (PR/CR) and non-responders including stable disease (SD) and progressive disease (PD) based on response evaluation criteria in solid tumors, RECIST v1.1. Wilcoxon test was used to analyze the differences between responders and non-responders under the TCE treatment using ggpubr package embedded in RStudio v1.2. The impact of sensitive parameters on the overall response rate was also studied with 95% Agresti-Coull confidence intervals (CIs) (31).

## Results

### TCE Module

#### Module Diagram

**Fig. 2:**
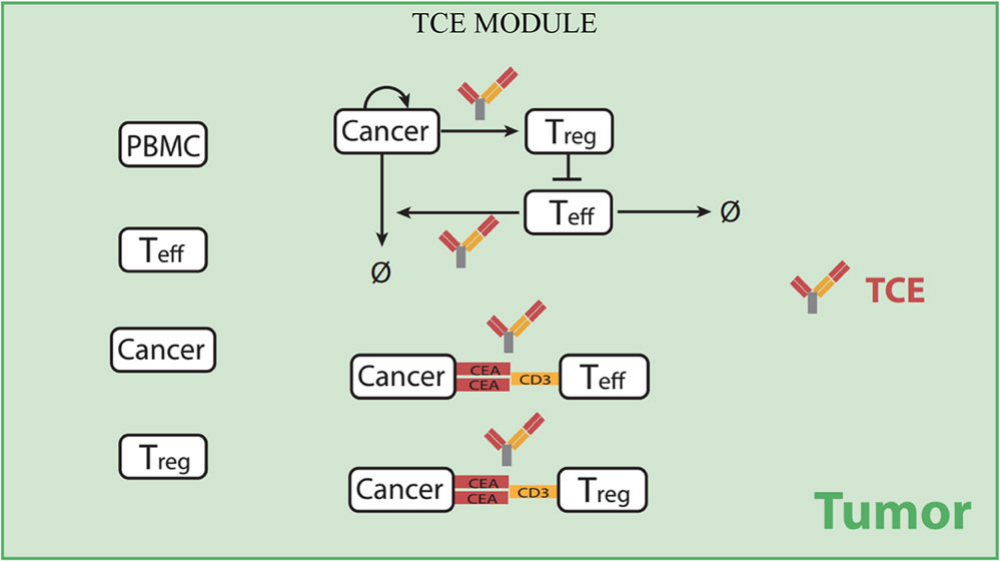
TCE module diagram. This diagram illustrates the interactions between TCEs, cancer cells, and T cells. T cells or peripheral blood mononuclear cells (PBMC) and cancer cells can be added based on *in vitro* experiment settings when using this module alone or determined by cancer cell growth rate, T cell transport, and antibody pharmacokinetic when combining with QSP model

Our previously published immuno-oncology QSP model (24) was extended by incorporating TCE dynamics as an independent module (Fig. 2). The governing equations for the module are presented in the Supplementary Information. Mature Teff, Treg, and TCEs are transported from the central compartment to the tumor compartment. Teff and Treg can be recruited by TCEs to retarget tumor cells in an MHC-independent pathway (32). TCE-induced activation of Teff was translated to cancer cell killing, and activation of Tregs was translated into Teff exhaustion using two distinct Hill equations (Supplementary Information Eqs. 9–12). As an independent module from the QSP model, it can be used to study the *in vitro* activity of TCEs.

#### Module Simulation Results

**Fig. 3:**
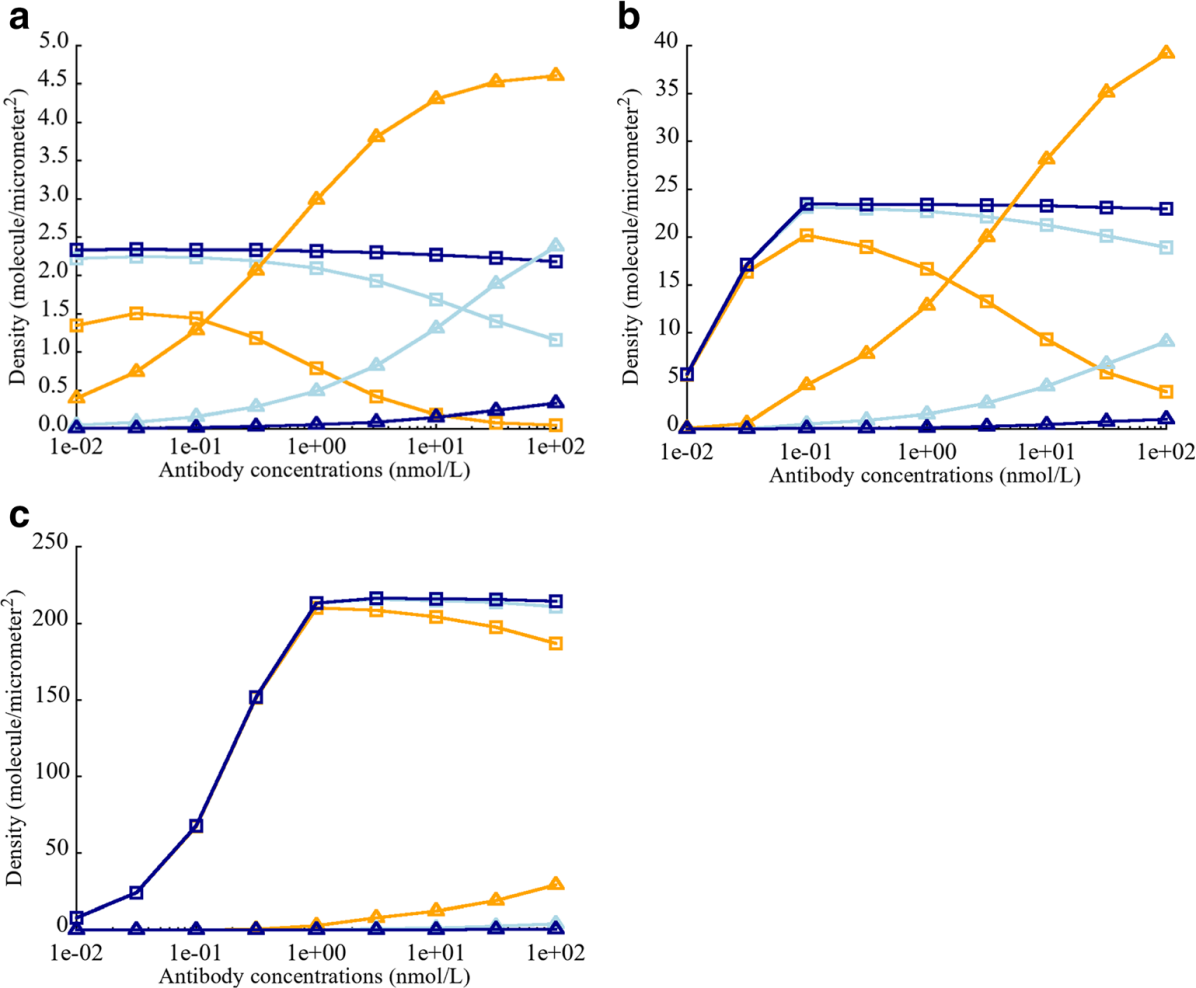
Effect of intrinsic cross-arm binding efficiency *λ* on biTTC and moTTC density when varying CEA expression on cancer cells. **a** CEA expression = 5E+3 sites/cell. **b** CEA expression = 5E+4 sites/cell. **c** CEA expression = 5E+5 sites/cell. biTTC density at *λ* = 1 (), moTTC density at *λ* = 1 (). biTTC density at *λ* = 100 (), moTTC density at *λ* = 100 (). biTTC density at *λ* = 10,000 (), moTTC density at *λ* = 10,000 ()

Bacac *et al.* reported mechanistic insights into the activity and mode of action of CEA-TCB; they found that tumor activity of CEA-TCB was positively correlated with CEA expression (10). The TCE module was calibrated against *in vitro* experimental results by adding 20 nM CEA-TCB to a constant number of PBMCs and tumor cells, using an E/T ratio of 10:1. The aim was to explore the effect of CEA expression on the Hill equation used to translate biTTC formation to TCE efficacy. Similar to the results reported by Bacac *et al.*, our model found that percent tumor lysis was significantly correlated to the number of CEA binding sites, and a threshold of 1.0E+4 sites/cell was required for tumor cell lysis (Fig. S2). MKN45, LS174T, HT-29, and CCD-841 cell lines, which have different levels of CEA expression, were also simulated, and the results showed similar trends in extent tumor lysis as reported by Bacac *et al.* (10) (Fig. S3). The trend of these four cell lines is in a good agreement to Bacac’s Fig. 3a except the cell line LS-174T. This difference may due to the experimental error since the measurement of tumor lysis was highly dependent on the time and release rate of lactate dehydrogenase (LDH). However, it has confirmed by their experiments that the high CEA expression always leads to better killing of cancer cell and the CEA-TCB always had very good killing of MKN45 cell line with the highest CEA expression in their study, which was consistent with our simulation results in Figs. S2 and S3. By fitting our module to the findings of Bacac *et al.*, we were able to estimate TCE concentration required for half-maximal activation, Hill coefficient, and intrinsic antibody cross-arm binding efficiency *λ*.

Once the Hill equation parameters were determined, we investigated the effects of several key parameters that may influence formation of biTTC and TCE potency, including intrinsic cross-arm binding efficiency *λ*, CEA expression, CEA-TCE binding affinity, and CD3-TCE binding affinity. An important assumption in this model is that the number of biTTC formed in the immunological synapse determines efficacy, since high-avidity binding to tumor targets can facilitate T cell activation. Formation of moTTC or other species such as CEA-TCE and CD3-TCE and binding to soluble CEA do not affect activity in this model.

#### Effect of CEA Expression on the Density of biTTC and moTTC

Different antibodies targeting CEA have different cross-arm binding affinities, thus varying parameter *λ*. Harms *et al.* have reported that the value of intrinsic cross-arm binding efficiency can significantly affect functional affinity in a context dependent manner (26). The affect of *λ* on biTTC formation was investigated by varying CEA expression from 5E+3 to 5E+5 sites/cell. The total number of CD3 binding sites (6.1E+4 sites/cell) was fixed (27–30). In the case of low or moderate CEA expression (5E+3 and 5E+4 binding sites/cell), *λ* was observed to have a large impact on biTTC density (Fig. 3a, b). Low levels of biTTC formation were observed for antibody concentration greater than 100 nM when *λ* was set equal to 1 or 100. However, biTTC formation was relatively insensitive to TCE concentration when *λ* was greater than 100. The sensitivity of biTTC formation to *λ* decreased as the number of CEA binding sites increased from 5E+3 to 5E+5 (Fig. 3). When CEA expression was high (5E+5 sites/cell), *λ* had minimal impact on biTTC density, for TCE concentrations ≤ 100 nM (Fig. 3c).

#### Effect of CEA-TCE Binding Affinity Kd on the Density of biTTC and moTTC

It is widely recognized that tumor antigen affinity is a key factor governing the potency of monoclonal antibodies, as well as TCEs. Higher affinity has been reported to show better potency (33–36). Bivalent binding results in much higher functional affinity between TCEs and tumor cells resulting in better potency (37–39). CEA binding affinity played an important role with respect to TCE activity and is an important factor for achieving high avidity. To assess the impact of *λ* and affinity on extent biTTC formation, CEA binding affinity was varied from 0.01 to 100 nM and CEA expression was fixed to 5E+4 binding sites/cell.

**Fig. 4:**
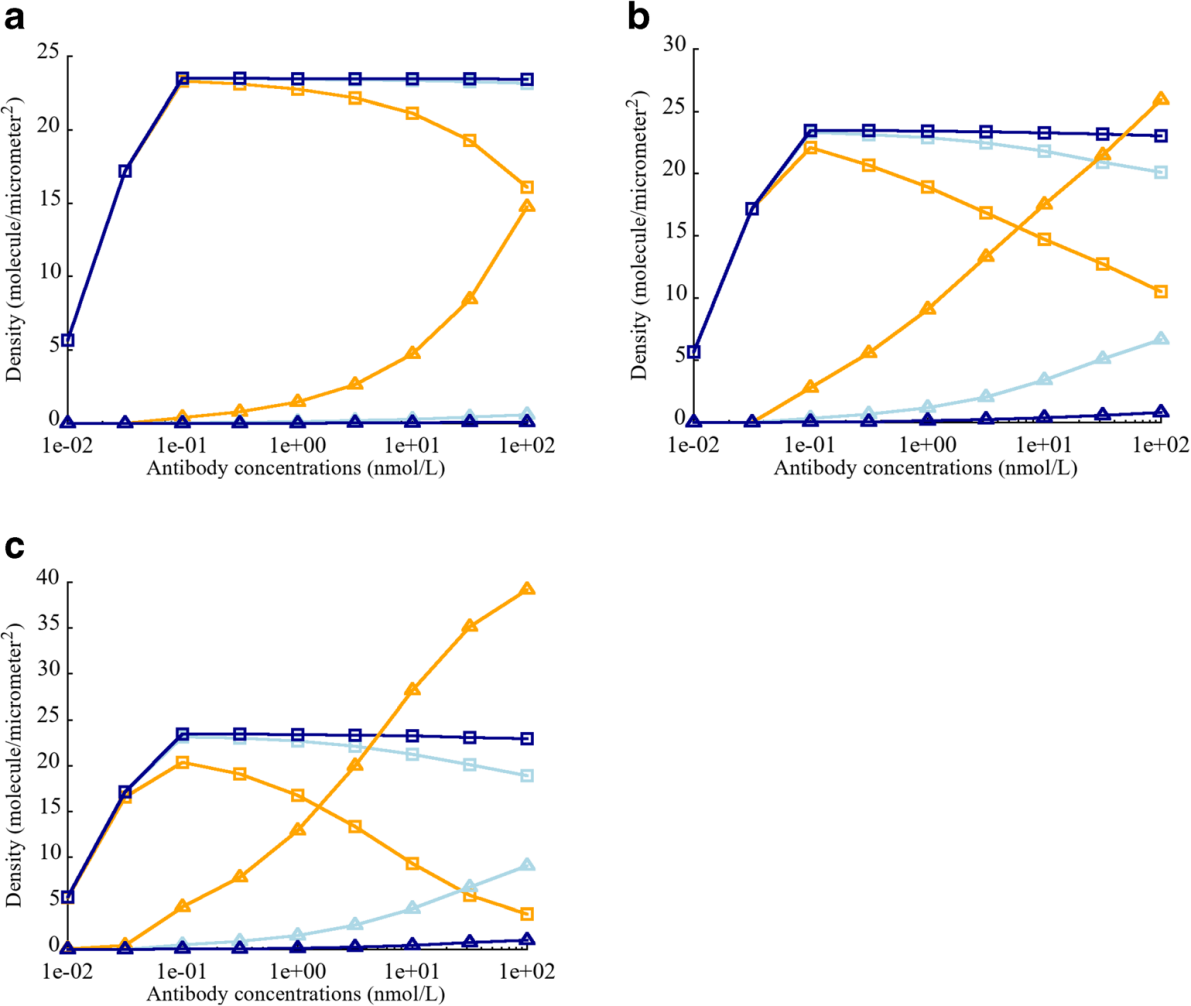
Effect of intrinsic cross-arm binding efficiency *λ* on biTTC and moTTC density when varying binding affinity Kd (CEA-TCE) **a** Kd = 0.01 nM. **b** Kd = 1 nM. **c** Kd = 100 nM. biTTC density at *λ* = 1 (), moTTC density at *λ* = 1 (). biTTC density at *λ* = 100 (), moTTC density at *λ* = 100 (). biTTC density at *λ* = 10,000 (), moTTC density at *λ* = 10,000 ()

When CEA monovalent binding affinity was high (0.01 nM), biTTC density was less sensitive to TCE concentration and *λ*, compared with weaker monovalent binding affinities (Fig. 4a). For intermediate (1 nM) and low (100 nM) monovalent CEA binding affinities, *λ* played key role in reducing the sensitivity of biTTC density to TCE concentration (Fig. 4b, c). This indicates that when designing TCEs with bivalent binding, monovalent tumor antigen affinity is unquestionably important; however, if this binding affinity does reach a threshold level, the assistance of a high *λ* is required.

#### Effect of CD3-TCE Binding Affinity Kd on the Density of biTTC and moTTC

**Fig. 5:**
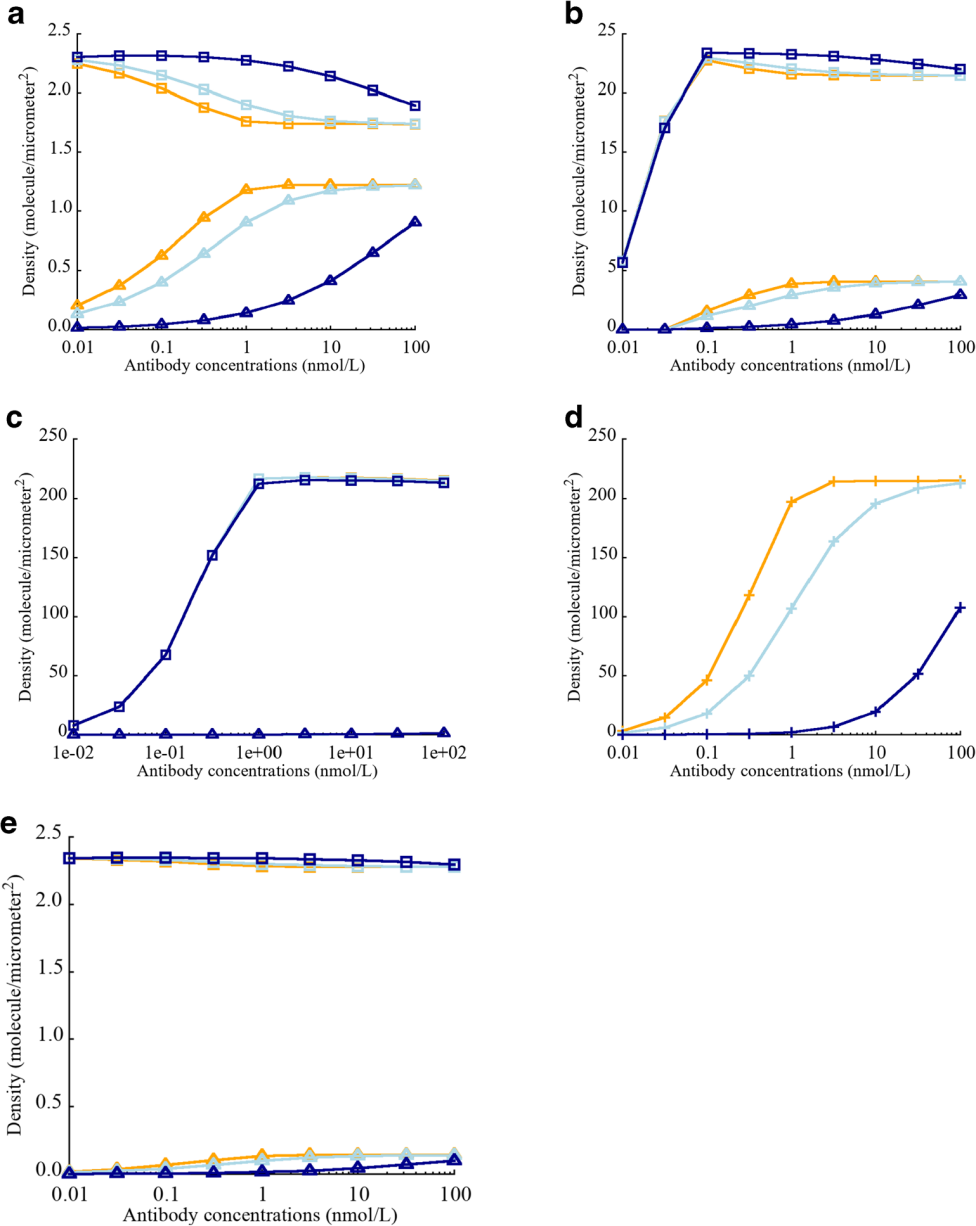
Effect of CD3 binding affinity Kd (CD3-TCE) on biTTC and moTTC density. **a** CEA expression = 5E+3 sites/cell. **b** CEA expression = 5E+4 sites/cell. **c** CEA expression = 5E+5 sites/cell. **d** Density of CD3-TCE dimer. **e** Effect of elevated *λ* (= 10,000) on the density of biTTC and moTTC. biTTC density at Kd = 0.01 (), moTTC density at Kd = 0.01 (). biTTC density at Kd = 1 (), moTTC density at Kd = 1 (). biTTC density at Kd = 100 (), moTTC density at Kd = 100 (). CD3-TCE dimer density at Kd = 0.01 (), 1 () and 100 nM ()

With few exceptions, TCEs are designed with monovalent CD3 binding to prevent T cell activation in the absence of tumor target engagement. The impact of CD3 affinity on TCE activity remains unclear, and published literature has shown conflicting results (34,40,41). The effect of varying CD3 affinity, with constant CEA affinity and avidity (anti-CEA = 130 nM and *λ* = 1000 for cibisatamab), showed that for low CEA expression (5E+3 and 5E+4 sites/cell), higher anti-CD3 affinity did not lead to higher biTTC density. On the contrary, low anti-CD3 affinity resulted in higher biTTC density, which corresponds to better potency (Fig. 5a, b). The reason for this counterintuitive behavior is that higher anti-CD3 affinity leads to the formation of more stable TCE-CD3 and CEA-TCE complexes, which impeded further generation of ternary complexes such as biTTC. This phenomenon was not observed for high CEA expression (5E+5 sites/cell) since there were more CEA binding sites available to form ternary structures with stable TCE-CD3 complexes (Fig. 5c). Increasing *λ* could reduce the effect CD3 affinity (Fig. 5e); however, since these parameters were known in this study for cibisatamab, and the impact of these parameters on the effects of TCEs was not the focus of this study, we will not discuss it in depth here. It is obvious that the effects of CD3 are complex and need to be carefully considered and tested when designing TCEs.

#### Bell-Shaped Concentration Relationship Observed for TCEs

**Fig. 6:**
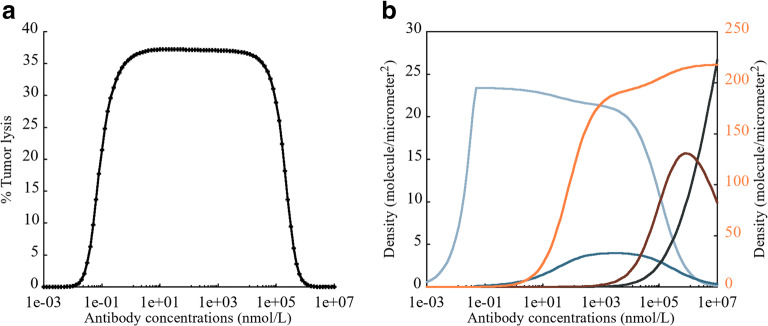
**a** Schematic of the bell-shaped concentration relationship observed for TCEs. **b** Primary axis: density of biTTC (), moTTC (), CEA-TCE dimer (), CEACEA-TCE trimer (); secondary *y*-axis: CD3-TCE dimer ()

The efficacy of TCEs is dependent on the extent of biTTC formation. A bell-shaped concentration-response relationship has been reported for other TCEs with monovalent tumor antigen binding (21). This phenomenon was shown to be true for bivalent binding TCEs, based on model simulations (Fig. 6a). The percentage of tumor lysis followed a bell-shaped relationship with a wide plateau, which was dependent on the formation of biTTC (light blue line in Fig. 6b). When the TCE concentration was low from 0.001 to 0.1 nM, biTTC density increased until reaching a peak around 0.1 nM. However, biTTC density slowly decreased when the TCE concentration increased from 0.1 to 10,000 nM, which did not significantly affect potency. When the concentration increases further beyond 10,000 nM, the density of other species increased significantly such as CEA-TCE dimer, CEA-CEA-TCE dimer, and CD3-TCE dimer, causing a significant drop in potency. However, the concentration ranges mentioned above depend on Kd, tumor-associated antigen expression, CD3 expression, and the ratio of cancer cells to Teff. Appropriate drug dosing regimen still requires detailed parameters of each drug and toxicological studies. Currently, we can only compute the changes in biTTC density following the increase in antibody concentration from a kinetic perspective.

## Quantitative Systems Pharmacology Model Including the TCE Module

The results from the first part proved the reliability of our TCE module to describe the *in vitro* dynamics of CEA-TCEs. After incorporating the TCE module into the previously described QSP model (24), we were able to predict the objective response for a virtual cohort of colorectal patients following cibisatamab monotherapy. RECIST category responses were calculated after a treatment period of 400 days when all patients who had PR/CR and SD almost reached convergence and their tumor size would not change anymore for a virtual cohort of 1750 patients that were created by sampling parameters within certain ranges based on clinical and experimental evidence. The baseline parameters of these virtual patients (Supplementary Information, Table S2) and their ranges (Supplementary Information, Table S3) are based on published literature. The ranges for parameters were chosen to be physiologically reasonable if experimental measurements are unavailable. Though we do not have the exact distribution of all parameters, we made an effort to ensure that the patients generated by the model are plausible. Those abnormal virtual patients with implausible physiological status were screened out and excluded; that was how we screened out 250/2000 patients, with 1750/2000 virtual patients remaining. A sensitivity analysis was performed for primary model parameters to identify the most influential ones. It is worth mentioning that we also varied the CEA-TCE and CD3-TCE binding affinity in order to study the effect of these parameters on the therapeutic effect, even though these parameters are known for cibisatamab. Although varying these two parameters will affect the overall response rate, the focus of the model is to find the most reliable predictive biomarkers rather than the overall response rate.

### Potential Biomarkers for TCE Monotherapy

**Fig. 7:**
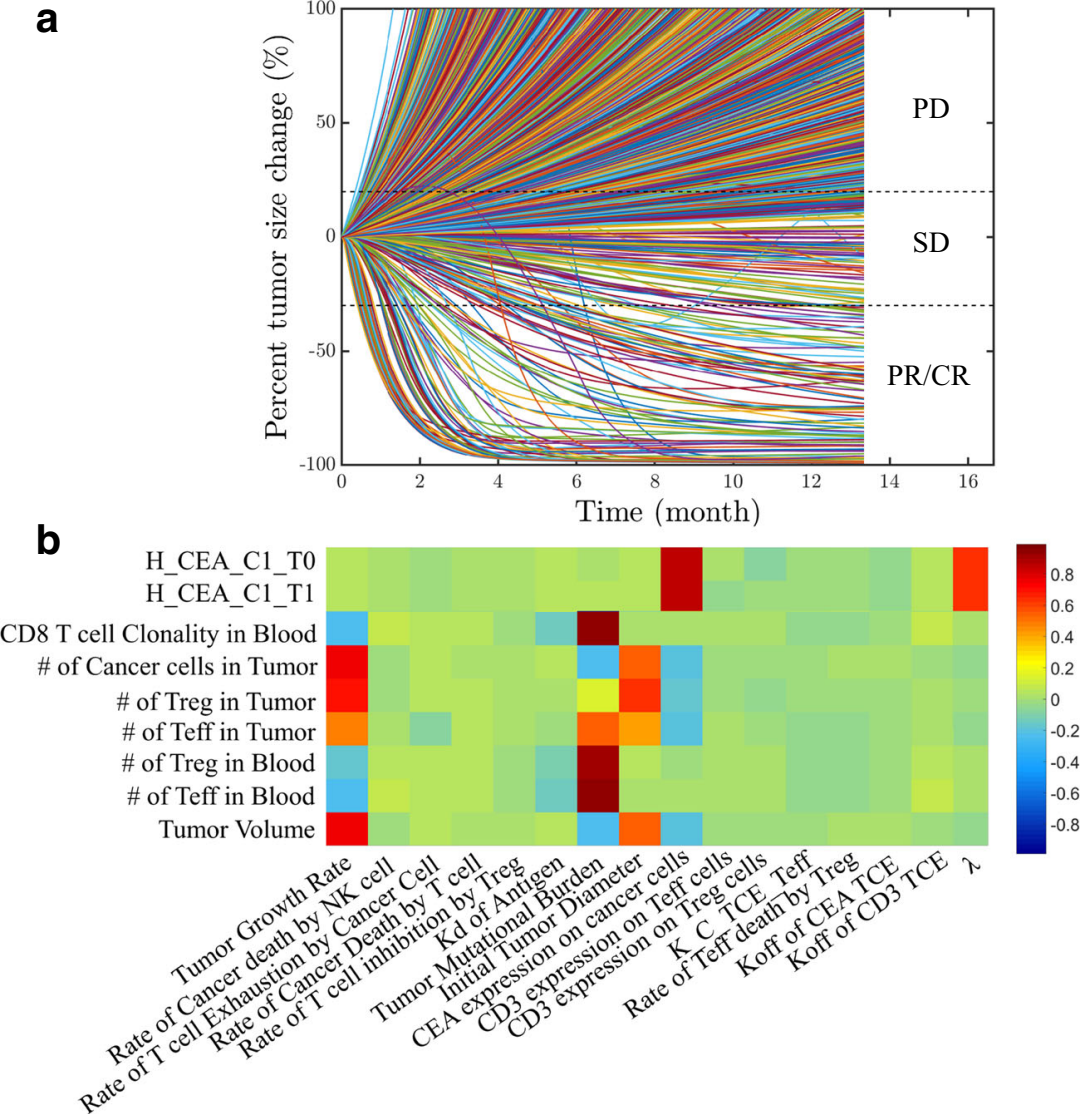

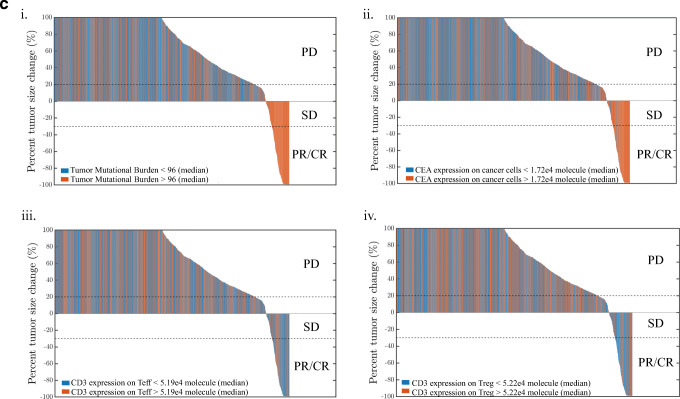
**a** Percent change in tumor size represented using RECIST criteria (a “spider” plot); note the diversity of the responses for different virtual patients. **b** The partial rank correlation coefficient (PRCC) for individual parameters. **c** (i) Waterfall plots for 1750 simulations while varying (i) TMB, (ii) CEA expression, (iii) CD3 expression on Teff cells, and (iv) CD3 expression on Treg cells

The overall response of cibisatamab monotherapy in colorectal cancer was investigated by simulating 2000 virtual humans characterized by different sets of model parameters. Parameters and their ranges are listed in the Supplementary Information. In the simulations, for each individual, the tumor starts growing from a single cell and continues to grow until it reaches a certain cell number or diameter. During this simulation, there were 1750 virtual patients whose tumor size reached the preset initial tumor diameter; the rest of them (i.e., 250) who did not develop tumors or with implausible physiological status were considered non-patients. These were considered the initial conditions for this cohort of virtual patients. From that point, we studied the patients’ response to cibisatamab monotherapy. Under conditions mimicking the NCT02324257 trial, virtual patients with metastatic colorectal cancer (mCRC) were treated with once weekly cibisatamab doses of 60–600 mg. We compared the overall response rates of all virtual patients treated with 60 mg or 600 mg cibisatamab and found no significant differences (< 1%), which was consistent with the clinical trial results. Thus, for subsequent simulations, we chose a 60-mg dose administered once weekly, after the tumor size reached its preset value. The simulated time-dependent percent tumor size changes are shown in Fig. 7a (spider plot) based on RECIST criteria (42). Among these patients, at 400 days, 130/1750 had PR/CR (7.4%), 123/1750 had SD (7.0%), and 1497/1750 had PD (85.5%). Note that the simulations show a number of cases where tumor growth was non-monotonic, i.e., the diameter initially increased and then tumor growth reversed and the tumor responded. Conversely, there were cases where the tumor initially responded, and then the tumor began to regrow. We note that the predicted percent response at present cannot be compared with clinical data since the only data reported were from a small and short-term clinical trial. In this trial, CT scans within days after the first dose showed 5% PR; 11% showed preliminary tumor size reduction, and at week 4–6, 28% of patients showed a metabolic partial response by PET scan (43). For example, in phase 1a and 1b, the response rates among 31 patients were reported as 6%, 39%, and 52% for PR/CR, SD, and PD, respectively. Thus, quantitative comparisons between the model-predicted and observed responses have to await data from larger clinical trials. Even then, it is possible that important parameters from treated patient will not be available such as distribution of all patients’ TMB, density of Teff and Treg in tumor, and expression of target antigens. Nevertheless, virtual patients in our model simulations can reflect a realistic parameter set. The main goal of simulating a larger patient sample size is to capture important biomarkers that may affect patient outcomes. We will further explore the variation of parameters within the simulated cohort below and show how these variations affect response rates (Fig. 9).

We investigated the sensitivity of predicted tumor volume against some of the parameters using partial rank correlation coefficient (PRCC), whose values range between − 1 (negative effect) and + 1 (positive effect) (44). The results are presented as a heatmap in Fig. 7b; the values of the PRCC are color-coded as indicated on the vertical strip. Tumor growth rate, TMB, patients’ initial tumor diameter, and CEA expression on cancer cells were significantly positively correlated to the tumor volume. We should point out that TMB in our model is defined as the number of activated T cell clones (24). This measure is related to the conventionally defined TMB as the number of mutations found in the DNA of cancer cells (45). In addition to tumor volume, tumor CEA expression and intrinsic cross-arm binding efficiency *λ* affected the TCE Hill equation, which is predicted to be an important factor for cibisatamab efficacy. These results explain how different parameter sets affect the immune response, resulting in distinct therapeutic effects. To better illustrate the effect of several sensitive parameters, the results are also shown as waterfall plots (Fig. 7c). Higher TMB and higher CEA expression on cancer cell corresponded to smaller tumor volume, indicating better efficacy (Fig. 7c (i), (ii)), whereas CD3 expression on Teff and Treg cells did not affect the outcome and no correlation was observed from the waterfall plot (Fig. 7c (iii), (iv)).

### Statistical Analysis for Non-responders and Responders

**Fig. 8:**
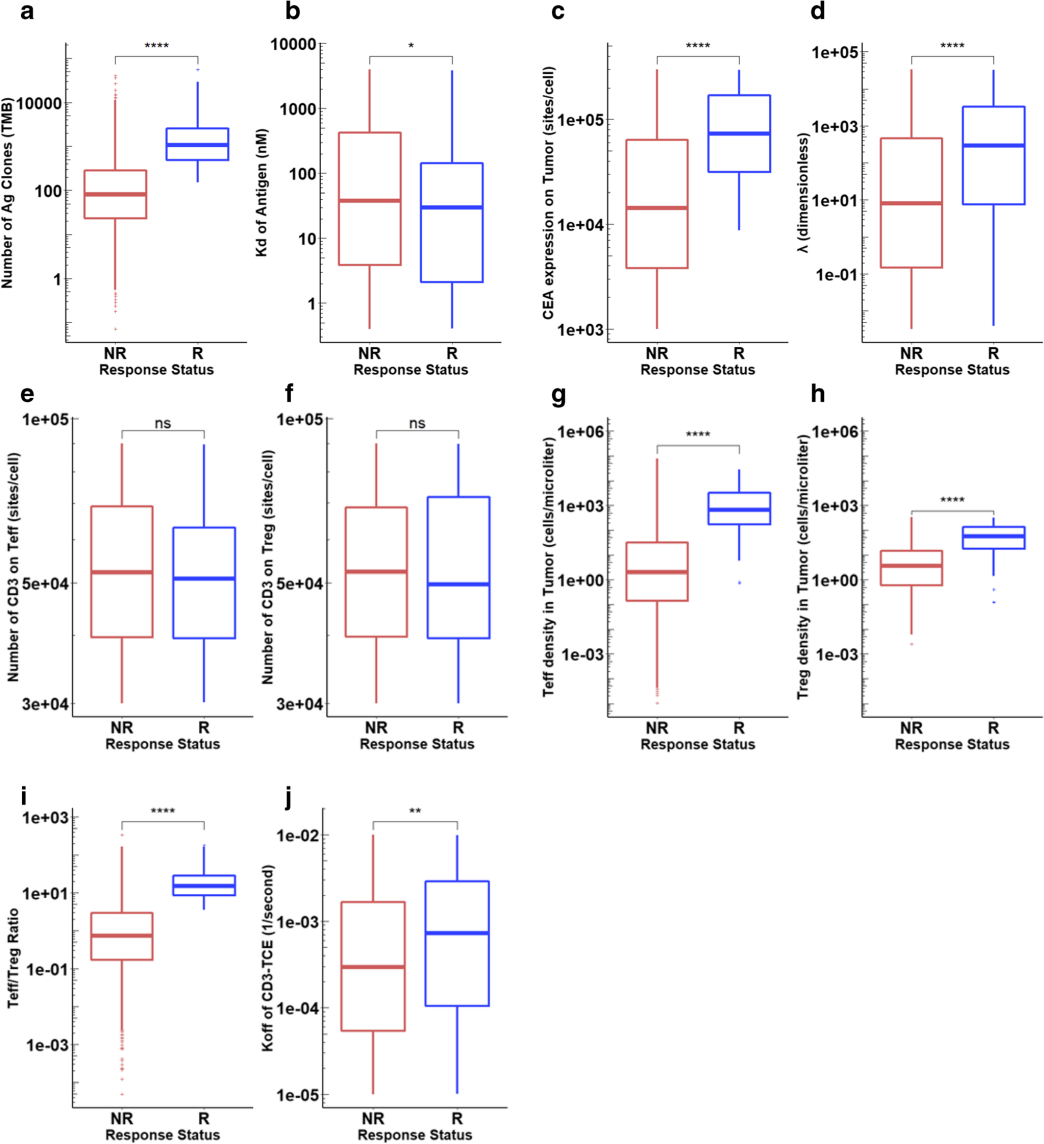
Distributions of potential biomarkers in NR and R. **a** TMB. **b** Kd of antigen. **c** CEA expression on tumor. **d***λ*. **e** CD3 expression on Treg. **f** CD3 expression on Teff. **g** Teff density in tumor. **h** Treg density in tumor. **i** Teff/Treg ratio in tumor. **j** koff of CD3-TCE

Due to the high sensitivity of tumor growth rate to TMB and CEA expression, the color of other sensitive parameters in PRCC was weakened so that it cannot be clearly seen in Fig. 7b. Statistical comparisons were conducted between the non-responders (SD and PD) and responders (PR/CR) to identify the most significant differences between them and to more clearly present the effect of other important parameters of interest. We carefully analyzed the distributions of TMB, MHC II antigen affinity, tumor CEA expression, intrinsic cross-arm binding efficiency *λ*, CD3 expression on Treg and Teff cells, Teff and Treg densities in tumor as well as their ratio (Teff/Treg), and CD3 binding affinity. Clearly TMB was significantly higher in responders than non-responders (Fig. 8a), whereas MHC II antigen affinity did not significantly affect the response (Fig. 8b). Responders had much higher CEA expression on cancer cells and stronger intrinsic cross-arm binding efficiency *λ*, leading to stable formation of biTTC (Fig. 8c, d). However, neither CD3 expression on Teff or Treg affected the response (Fig. 8e, f). Responders also had so-called hot tumors with high levels of both Teff and Treg infiltration (Fig. 8g, h). The Teff/Treg ratio was also higher in responders (Fig. 8l). Since the kon of CD3-TCE was set to a constant, changing koff represents a change in Kd of CD3-TCE. Although not particularly significant, higher koff (low affinity) instead corresponds to better response (Fig. 8i), which was consistent with the aforementioned *in vitro* results (“Effect of CD3-TCE binding affinity Kd on the density of biTTC and moTTC” section).

In order to more intuitively express the impact of these parameters on the overall response rate, we divided the 1750 virtual patients into several subgroups and then calculated the overall response rate with 95% confidence intervals for patients in these subgroups, with parameters above and below the median value (Table I). It is notable that the overall response rate of virtual patients who had TMB or Teff/Treg ratio lower than the median was 0%, which indicated that the overall response rate was highly dependent on the values of these two parameters. CEA expression, intrinsic cross-arm binding efficiency *λ*, CD3 binding affinity, and the density of Teff and Treg in tumor also lead to significantly different overall response rates. However, MHC II antigen affinity and CD3 expression on Teff and Treg had relatively little effect on the overall response rate.

**Table I:**
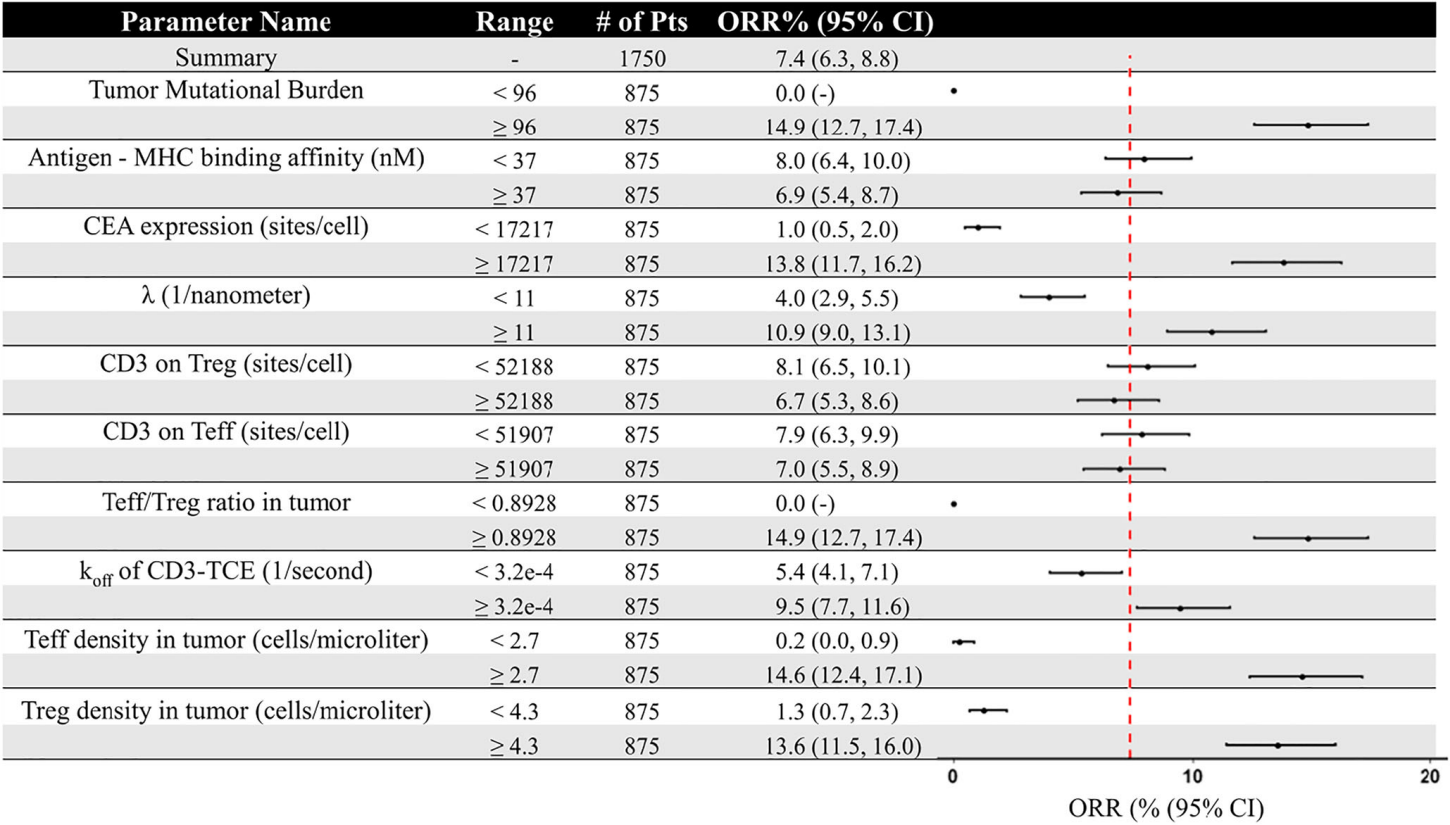
Response Rate in Subgroups of 1750 Virtual Patients

**Fig. 9:**
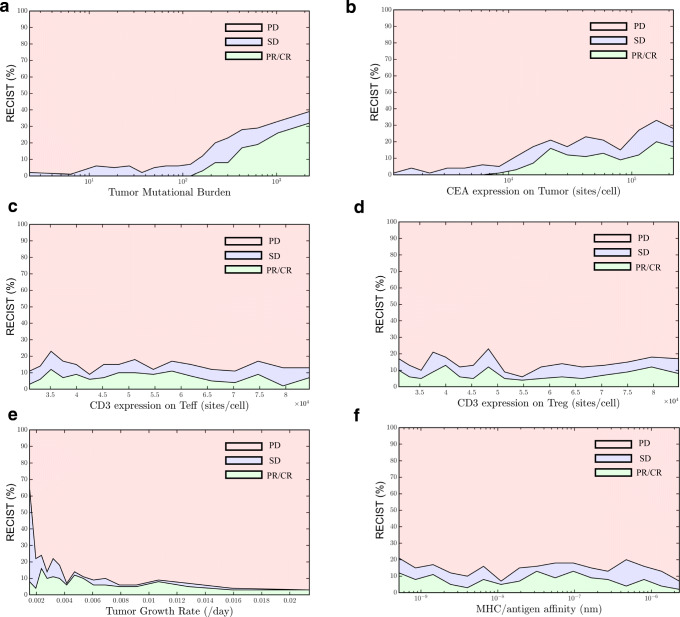
RECIST criteria vs. parameters. **a** TMB. **b** CEA expression on tumor. **c** CD3 expression on Teff. **d** CD3 expression on Treg. **e** Tumor growth rate. **f** MHC/antigen affinity

To gain insight into the impact of various parameters on how well cancer patients respond to cibisatamab treatment based on RECIST, all plausible virtual patients in our study were divided into different intervals according to the distribution of various parameters to calculate the overall response rate when the parameters were increasing; the results are presented in Fig. 9. The virtual patients are evenly divided into 17 subgroups, and within each subgroup are sorted by the selected parameter values in ascending order. The response status of each subgroup is plotted against the median parameter values in each subgroup. The number of patients falling in the PR/CR and SD region gradually increased with the increase of TMB and CEA expression on tumor cells (Fig. 9a, b) but did not change with CD3 expression (Fig. 9c, d). A negative correlation was found between patients with PR/CR or SD and tumor growth rate, indicating that faster tumor growth rates generally lead to poorer responses (Fig. 9e). Although patients’ subgroups with lower or higher MHC II antigen affinity showed similar overall response rate (Table I), a negative correlation was found between patients with PR/CR or SD and MHC II antigen affinity when MHC II antigen affinity was larger than 10^−7^ M, indicating that MHC II antigen affinity determines the efficiency of antigen-presenting cells and low MHC II antigen affinity (> 10^−7^ M) lead to poorer responses (Fig. 9f). This analysis provides rational hypotheses for the possible discrepancy between the outcome of clinical trials and the current simulations. Figure 9 shows a wider range of possible outcomes depending on the characteristics of patient cohorts.

## Discussion

T cell engagers, as a class of bispecific antibodies, have attracted a lot of attention recently due to their unique ability to activate cytotoxic T cells in an MHC-independent manner, thereby releasing cytokines and cytotoxic granules that inhibit tumor growth. Currently, two approved bispecific antibodies (one of the two is a TCE) are in clinical practice and about 100 are in clinical development (8,9). As an engineered antibody, there are many factors to consider carefully in the design process, such as the antibody format, target antigen on the T cell, tumor target antigen, and affinity and valency for both T cell and tumor antigens. These factors will ultimately determine TCE potency. These factors may be dependent on the cancer cell type, and there is currently a lack of effective ways to study the impact of changes in these factors. The TCE module described here provides a tool to solve this issue and provides a means for guiding the design of new TCEs, improving current TCEs, and can be used as an aid for designing and analyzing clinical trials.

Our TCE module has corroborated the importance of the biTCC formation for potent anticancer activity, and high-avidity is an important prerequisite for maintaining high biTCC density especially for those cells with low tumor antigen expression. Moderate to high monovalent binding affinity was also essential, since low affinity tumor antigen binding, even in the presence of high *λ*, resulted in suboptimal biTCC density. In addition to handling bivalent TCEs, our module can be readily modified to study monovalent TCEs by modifying the governing equations.

Optimization of CD3 binding affinity is another viable path for improving TCE potency. To avoid tumor target-antigen–independent T cell activation, monovalent binding to CD3 is preferred. Interestingly, increasing CD3 affinity was not beneficial for stable biTTC formation in this model. This finding is consistent with Bortoletto *et al.* who observed reduced T cell activation with increasing CD3 binding affinity (41). In other studies, a positive correlation between the tumor cell killing and CD3 binding affinity was also reported by Ellwanger *et al.* using different anti-CD3 antibodies, target antigens, and formats (34). High anti-CD3 binding affinity has been associated with preferential distribution to CD3-rich lymphoid tissues and less effective tumor distribution. Optimization of CD3 affinity needs to consider many factors, and our model can provide some aspects of the impact of anti-CD3 affinity on drug efficacy (46).

The relationship between biTTC density and antibody concentration can also be monitored by our model under the influence of various factors. The amount of all TCE-related species can be monitored to study how they change with increasing TCE concentration. For TCEs with fixed parameters, our model can quickly find a reasonable range of antibody concentrations for optimal efficacy. When designing bispecific antibodies, our model can also be used to predict the appropriate range of binding parameters to support an optimal dose regimen, especially when considering combination therapies.

In addition to providing theoretical guidance for molecular design using the newly developed TCE module, our previously published QSP models demonstrated the ability to model tumor immunity and response to immune checkpoint inhibitors (16,18,24). The models were also used for biomarker discovery and performing virtual clinical trials by integrating anti-tumor immune response, antigen processing/presentation, and T cell priming in lymph nodes. The validated model provided a starting point for the study of TCEs. We integrated the new TCE module with the whole QSP model while keeping the main QSP model unchanged, enabling a virtual clinical trial to be performed for cibisatamab in colorectal cancer patients. All simulations started from a single cancer cell, thereby capturing individual initial conditions before cibisatamab treatment. Progression of the tumor was then simulated over the next 400 days to observe tumor size changes and identify potential biomarkers.

TMB was one of the most important predictive biomarkers affecting tumor size. It directly affects the expansion of Teff cells and higher TMB corresponds to higher T cell density and Teff/Treg ratio in tumor. As predicted by our model, low TMB corresponded to a low response rate. There was no responder found when either TMB or Teff/Treg ratio was below the median. Other potential biomarkers such as CEA expression and intrinsic cross-arm binding efficiency *λ* showed a positive correlation with overall response rates. Interestingly, patient cohorts with different CEA expression levels showed different sensitivity to *λ*. For patients with intermediate CEA expression (1E+4–3E+5 sites/cell), *λ* has significant impact on tumor diameter, since *λ* ensured the formation of stable biTTC for cells with lower CEA expression; for high CEA expression cells (3E+5–1E+6 sites/cell), the sensitivity was reduced since the effect of *λ* on high expression tumor cells was moderate (Fig. S4A,B). These observations are also consistent with the results in “Effect of CEA expression on the density of biTTC and moTTC” section, i.e., *λ* had little effect on cells with high tumor target antigen expression. Anti-CD3 affinity (koff of CD3-TCE) was negatively correlated with overall response rates. These are consistent with the predictions of the stand-alone TCE module.

CD3 expression on Teff and Treg cells was based on available experimental measurements; our model limited the CD3 expression to the data reported in the literature and within this range had little effect on TCE efficacy. Importantly, it has been reported that Treg cells can also be activated by TCEs and have the potential to inhibit the activity of Teff cells, even though the overall response rate was highly dependent on the Teff/Treg ratio. However, our model predicted that the impact of Treg on Teff cells can be ignored. The reason being that there are three main ways of T cell exhaustion including basic apoptosis of T cells, additional death caused by Treg and cancer cells. Typically, non-responders had faster tumor growth rate resulting in larger tumor with more cancer cells. These cancer cells would kill more Teff through several pathways. The death of Teff by Treg may not be able to show significant impact on the number of Teff, but the overall death rate of Teff was a very sensitive parameter, which corresponded to Teff density in tumor and Teff/Treg ratio. Other related reports also pointed out that CD8+ T cells respond much faster by cross-linking via bispecific antibodies than do CD4+ T cells and many other studies have shown successful treatment with bispecific antibodies in the presence of Tregs (9,12,46,47). It has been reported that E/T ratio changed the rate of T cell killing activity (48–50). T cell killing activity may have been saturated with high E/T ratio (10:1), which would result in underestimation of both T cell activity and TCE efficiency. Thus, we have taken the uncertainty of it into consideration by adding the T cell killing activity to the list of sensitivity analysis parameters to explore the effect of underlying underestimation of T cell killing activity. Within the range we defined for T cell activity (Table S3, Rate of cancer death by T cell), it did not show significant effect on the treatment outcomes (Fig. 7b).

By assessing the tumor response using RECIST vs. a set of parameters, we can more intuitively assess the impact of a parameter on the patients’ response. The increase in some parameters is positively correlated with treatment effect while others are negatively correlated. Interestingly, for some parameters, once they exceed a threshold value, the patients’ overall response rate will significantly increase, and further increases in these parameters has little effect on the overall response rate. An example of such behavior is the CEA expression on tumor cell, where a poor response was found for those patients with low CEA expression (< 1.0E+4 sites/cell). For patients with higher CEA expression (> 1.0E+4 sites/cell), the overall response rate was enhanced significantly, but no correlation was found between CEA expression and overall response rate above this level. Patients with CEA expression ranging from 1.0E+4 to 3.0E+5 have a similar overall response rate of ~ 12%.

Enhanced antitumor activity of cibisatamab in combination with anti-PD-L1 antibody atezolizumab has been reported in phase 1a and 1b studies (clinical trials: NCT02324257 and NCT02650713). The current TCE-extended QSP model can be used to identify potential predictive biomarkers for such combinations by comprehensive analysis of patients’ response and the differences between the non-responders and responders. Increased emphasis on the clinical development of monoclonal and bispecific antibodies that harness a patient’s own immune system to kill cancer cells necessitates the availability of highly parameterized QSP models that can be used to optimize clinical study design and the selection of combination partners for oncology targets. Our modular model makes the process of simulating virtual clinical trials faster and easier, by enabling the incorporation of additional modules as new immune mechanisms are discovered.

Even though we have calibrated the TCE module using experimental data, there are still some factors that have not been considered completely. In the current model, one assumption is that moTTC does not contribute significantly to the activation of T cells. Because of their lower affinity and stability compared with biTTC, their effects were ignored as a first approximation; however, further validation is needed. Tregs have been reported to have potent cytotoxic effects through the granzyme-perforin pathway (47). The death of cancer cells by Treg needs to be added and calibrated based on available experiments. Currently the TCE module only applies to bivalent TCEs. The dynamics of monovalent TCEs needs to be modified and related parameters also need to be recalibrated. The numbers of naïve CD8+, and CD4+ T cells are constant in central and tumor-draining lymph node compartments. Dynamics of the naïve T cells is important in large tumors in which T cell activation could result in depletion of antigen-specific naïve T cells and in turn cause non-response. The assumption of constant density of naïve T cells could hold true for smaller tumors but most likely will overestimate the response in large tumors. In our next version of the model, we will modify the model by incorporating naïve T cell dynamics including the naïve T cell selection in thymus, proliferation in the peripheral tissues as well as differentiation and death. Importantly, this whole QSP model has limitations in predicting the overall response rates of patients since we cannot accurately estimate the range and distribution of patient parameters and the estimation is highly dependent on the clinical measurements. Certain parameters are difficult to measure in the clinic, and their values have to be extrapolated from animal experiments, which will affect the accuracy of the predicted overall response rate and the reliability of the relevant biomarkers. The problem of how to generate virtual patients and virtual cohorts is an area of active research in academia and pharmaceutical industry (14,51–53), and more reliable ways should emerge in the future to improve model predictions. We also envision that the predictive power of QSP modeling will increase with each application in different cancer types (54,55).

## Conclusion

We extended the application of a whole-patient QSP model by adding a TCE module and applied the model to TCE monotherapy in colorectal cancer. The newly added module was calibrated based on the available *in vitro* data on over 100 colorectal cancer cell lines. It has been shown to be able to reproduce the *in vitro* experimental data and the effect of some parameters has been determined by varying them in our model. By studying the overall response rate of a cohort of virtual colorectal patients, we were able to determine the most influential and sensitive parameters and quantified their impact on overall tumor response rates. The TCE module can be used to help design new antibodies and provide instant information on the impact of various parameters on antibody properties and their dynamics. Once more, accurate parameters from the patient populations become available from future clinical trials such as their TMB, distribution of tumor-associated antigens’ expression, Teff density in tumor as well as Treg. The model can be recalibrated to improve the predictions of patients’ overall response rate based on their individual characteristics. In addition, our TCE module as well as the whole QSP model can be readily extended to other novel antibodies and cancer types, as long as the corresponding parameters are amended.

## Electronic Supplementary Material

ESM 1 (DOCX 448 kb)

## References

[1] Ciardiello D, Vitiello PP, Cardone C, Martini G, Troiani T, Martinelli E, Ciardiello F (2019). Immunotherapy of colorectal cancer: challenges for therapeutic efficacy. Cancer Treat Rev.

[2] Dawson H, Kirsch R, Messenger D, Driman D (2019). A review of current challenges in colorectal cancer reporting. Arch Pathol Lab Med.

[3] Tang J, Yu JX, Lin YQ (2018). The clinical trial landscape for PD1/PDL1 immune checkpoint inhibitors. Nat Rev Drug Discov.

[4] Gadiot J, Hooijkaas AI, Kaiser ADM, van Tinteren H, van Boven H, Blank C (2011). Overall survival and PD-L1 expression in metastasized malignant melanoma. Cancer..

[5] Chae YK, Arya A, Iams W, Cruz MR, Chandra S, Choi J, Giles F (2018). Current landscape and future of dual anti-CTLA4 and PD-1/PD-L1 blockade immunotherapy in cancer; lessons learned from clinical trials with melanoma and non-small cell lung cancer (NSCLC). J Immunother Cancer.

[6] Cantero-Cid R, Casas-Martin J, Hernandez-Jimenez E, Cubillos-Zapata C, Varela-Serrano A, Avendano-Ortiz J (2018). PD-L1/PD-1 crosstalk in colorectal cancer: are we targeting the right cells?. BMC Cancer.

[7] Yaghoubi N, Soltani A, Ghazvini K, Hassanian SM, Hashemy SI (2019). PD-1/ PD-L1 blockade as a novel treatment for colorectal cancer. Biomed Pharmacother.

[8] Labrijn AF, Janmaat ML, Reichert JM, Parren P (2019). Bispecific antibodies: a mechanistic review of the pipeline. Nat Rev Drug Discov.

[9] Yu SN, Li AP, Liu Q, Yuan X, Xu HX, Jiao DC, Pestell RG, Han X, Wu K (2017). Recent advances of bispecific antibodies in solid tumors. J Hematol Oncol.

[10] Bacac M, Fauti T, Sam J, Colombetti S, Weinzierl T, Ouaret D, Bodmer W, Lehmann S, Hofer T, Hosse RJ, Moessner E, Ast O, Bruenker P, Grau-Richards S, Schaller T, Seidl A, Gerdes C, Perro M, Nicolini V, Steinhoff N, Dudal S, Neumann S, von Hirschheydt T, Jaeger C, Saro J, Karanikas V, Klein C, Umana P (2016). A novel carcinoembryonic antigen T-cell bispecific antibody (CEA TCB) for the treatment of solid tumors. Clin Cancer Res.

[11] Lehmann S, Perera R, Grimm HP, Sam J, Colombetti S, Fauti T, Fahrni L, Schaller T, Freimoser-Grundschober A, Zielonka J, Stoma S, Rudin M, Klein C, Umana P, Gerdes C, Bacac M (2016). In vivo fluorescence imaging of the activity of CEA TCB, a novel T-cell bispecific antibody, reveals highly specific tumor targeting and fast induction of T-cell-mediated tumor killing. Clin Cancer Res.

[12] Herrmann I, Baeuerle PA, Friedrich M, Murr A, Filusch S, Ruttinger D (2010). Highly efficient elimination of colorectal tumor-initiating cells by an EpCAM/CD3-bispecific antibody engaging human T cells. PLoS One.

[13] Yiu AJ, Yiu CY (2016). Biomarkers in colorectal cancer. Anticancer Res.

[14] Cheng YG, Thalhauser CJ, Smithline S, Pagidala J, Miladinov M, Vezina HE, Gupta M, Leil TA, Schmidt BJ (2017). QSP toolbox: computational implementation of integrated workflow components for deploying multi-scale mechanistic models. Aaps J.

[15] Kirouac DC (2018). How do we "validate" a QSP model?. CPT Pharmacometrics Syst Pharmacol.

[16] Milberg O, Gong C, Jafarnejad M, Bartelink IH, Wang B, Vicini P, Narwal R, Roskos L, Popel AS (2019). A QSP model for predicting clinical responses to monotherapy, combination and sequential therapy following CTLA-4, PD-1, and PD-L1 checkpoint blockade. Sci Rep.

[17] Norton KA, Gong C, Jamalian S, Popel AS (2019). Multiscale agent-based and hybrid modeling of the tumor immune microenvironment. Processes..

[18] Wang HW, Milberg O, Bartelink IH, Vicini P, Wang B, Narwal R, Roskos L, Santa-Maria CA, Popel AS (2019). In silico simulation of a clinical trial with anti-CTLA-4 and anti-PD-L1 immunotherapies in metastatic breast cancer using a systems pharmacology model. R Soc Open Sci.

[19] Bai JPF, Earp JC, Pillai VC (2019). Translational quantitative systems pharmacology in drug development: from current landscape to good practices. Aaps J.

[20] Bradshaw EL, Spilker ME, Zang R, Bansal L, He H, Jones RDO, le K, Penney M, Schuck E, Topp B, Tsai A, Xu C, Nijsen MJMA, Chan JR (2019). Applications of quantitative systems pharmacology in model-informed drug discovery: perspective on impact and opportunities. CPT Pharmacometrics Syst Pharmacol.

[21] Betts A, Haddish-Berhane N, Shah DK, van der Graaf PH, Barletta F, King L, Clark T, Kamperschroer C, Root A, Hooper A, Chen X (2019). A translational quantitative systems pharmacology model for CD3 bispecific molecules: application to quantify T cell-mediated tumor cell killing by P-cadherin LP DART((R)). Aaps J.

[22] Yuraszeck T, Bartlett D, Singh I, Reed M, Pagano S, Zhu M (2016). A quantitative systems pharmacology (QSP) model to assess the action of blinatumomab in NHL patients (pts). J Clin Oncol.

[23] Demin O, Nikitich A, Demin O (2019). Quantitative systems pharmacology modeling of immunotherapies in B-cell acute lymphoblastic leukemia. Cancer Res.

[24] Jafarnejad M, Gong C, Gabrielson E, Bartelink IH, Vicini P, Wang B, Narwal R, Roskos L, Popel AS (2019). A computational model of neoadjuvant PD-1 inhibition in non-small cell lung cancer. Aaps J.

[25] Koristka S, Cartellieri M, Arndt C, Feldmann A, Seliger B, Ehninger G, *et al*. Tregs activated by bispecific antibodies killers or suppressors? Oncoimmunology. 2015;4(3):e994441. 10.4161/2162402X.2014.994441 PMC4404924 25949920

[26] Harms BD, Kearns JD, Iadevaia S, Lugovskoy AA (2014). Understanding the role of cross-arm binding efficiency in the activity of monoclonal and multispecific therapeutic antibodies. Methods..

[27] El Hentati FZ, Gruy F, Iobagiu C, Lambert C (2010). Variability of CD3 membrane expression and T cell activation capacity. Cytometry B Clin Cytom.

[28] Ginaldi L, Farahat N, Matutes E, DeMartinis M, Morilla R, Catovsky D (1996). Differential expression of T cell antigens in normal peripheral blood lymphocytes: a quantitative analysis by flow cytometry. J Clin Pathol.

[29] Thibault G, Bardos P (1995). Compared TCR and CD3-epsilon expression on alpha-beta and gamma-delta t-cells - evidence for the association of 2 TCR heterodimers with 3 CD3-epsilon chains in the TCR/CD3 complex. J Immunol.

[30] Nicolas L, Monneret G, Debard AL, Blesius A, Gutowski MC, Salles G, Bienvenu J (2001). Human gamma delta T cells express a higher TCR/CD3 complex density than alpha beta T cells. Clin Immunol.

[31] Brown LD, Cai TT, DasGupta A (2002). Confidence intervals for a binomial proportion and asymptotic expansions. Ann Stat.

[32] Koristka S, Cartellieri M, Theil A, Feldmann A, Arndt C, Stamova S, Michalk I, Töpfer K, Temme A, Kretschmer K, Bornhäuser M, Ehninger G, Schmitz M, Bachmann M (2012). Retargeting of human regulatory T cells by single-chain bispecific antibodies. J Immunol.

[33] Root AR, Cao W, Li BL, LaPan P, Meade C, Sanford J, Jin M, O’Sullivan C, Cummins E, Lambert M, Sheehan A, Ma W, Gatto S, Kerns K, Lam K, D’Antona A, Zhu L, Brady W, Benard S, King A, He T, Racie L, Arai M, Barrett D, Stochaj W, LaVallie E, Apgar J, Svenson K, Mosyak L, Yang Y, Chichili G, Liu L, Li H, Burke S, Johnson S, Alderson R, Finlay W, Lin L, Olland S, Somers W, Bonvini E, Gerber HP, May C, Moore P, Tchistiakova L, Bloom L (2016). Development of PF-06671008, a highly potent anti-P-cadherin/anti-CD3 bispecific DART molecule with extended half-life for the treatment of cancer. Antibodies..

[34] Ellwanger K, Reusch U, Fucek I, Knackmuss S, Weichel M, Gantke T, Molkenthin V, Zhukovsky EA, Tesar M, Treder M (2017). Highly specific and effective targeting of EG FRvIII-positive tumors with TandAb antibodies. Front Oncol.

[35] Alvarez-Cienfuegos A, Nunez-Prado N, Compte M, Cuesta AM, Blanco-Toribio A, Harwood SL (2016). Intramolecular trimerization, a novel strategy for making multispecific antibodies with controlled orientation of the antigen binding domains. Sci Rep.

[36] Harwood SL, Alvarez-Cienfuegos A, Nunez-Prado N, Compte M, Hernandez-Perez S, Merino N (2018). ATTACK, a novel bispecific T cell-recruiting antibody with trivalent EGFR binding and monovalent CD3 binding for cancer immunotherapy. Oncoimmunology..

[37] Bacac M, Klein C, Umana P (2016). CEA TCB: A novel head-to-tail 2:1 T cell bispecific antibody for treatment of CEA-positive solid tumors. Oncoimmunology.

[38] Bacac M, Umana P, Herter S, Colombetti S, Sam J, Le Clech M (2016). CD20 Tcb (RG6026), a novel "2:1" T cell bispecific antibody for the treatment of B cell malignancies. Blood..

[39] Bacac M, Colombetti S, Herter S, Sam J, Perro M, Chen S, Bianchi R, Richard M, Schoenle A, Nicolini V, Diggelmann S, Limani F, Schlenker R, Hüsser T, Richter W, Bray-French K, Hinton H, Giusti AM, Freimoser-Grundschober A, Lariviere L, Neumann C, Klein C, Umaña P (2018). CD20-TCB with obinutuzumab pretreatment as next-generation treatment of hematologic malignancies. Clin Cancer Res.

[40] Reusch U, Duell J, Ellwanger K, Herbrecht C, Knackmuss SHJ, Fucek I, Eser M, McAleese F, Molkenthin V, le Gall F, Topp M, Little M, Zhukovsky EA (2015). A tetravalent bispecific TandAb (CD19/CD3), AFM11, efficiently recruits T cells for the potent lysis of CD19(+) tumor cells. MAbs..

[41] Bortoletto N, Scotet E, Myamoto Y, D’Oro U, Lanzavecchia A (2002). Optimizing anti-CD3 affinity for effective T cell targeting against tumor cells. Eur J Immunol.

[42] Eisenhauer EA, Therasse P, Bogaerts J, Schwartz LH, Sargent D, Ford R, Dancey J, Arbuck S, Gwyther S, Mooney M, Rubinstein L, Shankar L, Dodd L, Kaplan R, Lacombe D, Verweij J (2009). New response evaluation criteria in solid tumours: revised RECIST guideline (version 1.1). Eur J Cancer.

[43] Tabernero J, Melero I, Ros W, Argiles G, Marabelle A, Rodriguez-Ruiz ME, Albanell J, Calvo E, Moreno V, Cleary JM, Eder JP, Karanikas V, Bouseida S, Sandoval F, Sabanes D, Sreckovic S, Hurwitz H, Paz-Ares LG, Saro Suarez JM, Segal NH (2017). Phase Ia and lb studies of the novel carcinoembryonic antigen (CEA) 1-cell bispecific (CEA CD3 TCB) antibody as a single agent and in combination with atezolizumab: preliminary efficacy and safety in patients with metastatic colorectal cancer (mCRC). J Clin Oncol.

[44] Marino S, Hogue IB, Ray CJ, Kirschner DE (2008). A methodology for performing global uncertainty and sensitivity analysis in systems biology. J Theor Biol.

[45] Jia QZ, Wu W, Wang YQ, Alexander PB, Sun CD, Gong ZH, Cheng JN, Sun H, Guan Y, Xia X, Yang L, Yi X, Wan YY, Wang H, He J, Futreal PA, Li QJ, Zhu B (2018). Local mutational diversity drives intratumoral immune heterogeneity in non-small cell lung cancer. Nat Commun.

[46] Ellerman D (2019). Bispecific T-cell engagers: towards understanding variables influencing the in vitro potency and tumor selectivity and their modulation to enhance their efficacy and safety. Methods..

[47] Choi BD, Gedeon PC, Herndon JE, Archer GE, Reap EA, Sanchez-Perez L, Mitchell DA, Bigner DD, Sampson JH (2013). Human regulatory T cells kill tumor cells through granzyme-dependent cytotoxicity upon retargeting with a bispecific antibody. Cancer Immunol Res.

[48] Vasconcelos Z, Muller S, Guipouy D, Yu W, Christophe C, Gadat S (2015). Individual human cytotoxic T lymphocytes exhibit intraclonal heterogeneity during sustained killing. Cell Rep.

[49] Halle S, Halle O, Forster R (2017). Mechanisms and dynamics of T cell-mediated cytotoxicity in vivo. Trends Immunol.

[50] Thompson ED, Enriquez HL, Fu YX, Engelhard VH (2010). Tumor masses support naive T cell infiltration, activation, and differentiation into effectors. J Exp Med.

[51] Allen RJ, Rieger TR, Musante CJ (2016). Efficient generation and selection of virtual populations in quantitative systems pharmacology models. CPT Pharmacometrics Syst Pharmacol.

[52] Rieger TR, Allen RJ, Bystricky L, Chen YZ, Colopy GW, Cui YF, Gonzalez A, Liu Y, White RD, Everett RA, Banks HT, Musante CJ (2018). Improving the generation and selection of virtual populations in quantitative systems pharmacology models. Prog Biophys Mol Biol.

[53] Perlstein D, Shlagman O, Kogan Y, Halevi-Tobias K, Yakobson A, Lazarev I, Agur Z (2019). Personal response to immune checkpoint inhibitors of patients with advanced melanoma explained by a computational model of cellular immunity, tumor growth, and drug. PLoS One.

[54] Wang HW, Sove RJ, Jafarnejad M, Rahmeh S, Jaffee EM, Stearns V, *et al*. Conducting a virtual clinical trial in HER2-negative breast cancer using a quantitative systems pharmacology model with an epigenetic modulator and immune checkpoint inhibitors. Front Bioeng Biotechnol. 2020;8:141. 10.3389/fbioe.2020.00141 PMC7051945 32158754

[55] Zhao C, Mirando AC, Sove RJ, Medeiros TX, Annex BH, Popel AS. A mechanistic integrative computational model of macrophage polarization: implications in human pathophysiology. PLoS Comp Biol. 2019;15(11):e1007468. 10.1371/journal.pcbi.1007468 PMC6860420 31738746

