# Supplementary material for: A Quantitative Systems Pharmacology Model of T Cell Engager Applied to Solid Tumor
Source: AAPS J. 2020 Jun 12;22(4):85. doi: 10.1208/s12248-020-00450-3 (PMC7293198; doi:10.1208/s12248-020-00450-3)
Supplement: Supplementary file 1 — (DOCX 448 kb) [file 12248_2020_450_MOESM1_ESM.docx]

**A Quantitative Systems Pharmacology model of T cell engager applied to solid tumor**

Huilin Ma^1^, Hanwen Wang^1^, Richard J. Sove^1^, Mohammad Jafarnejad^1^, Chia-Hung Tsai^3^, Jun Wang^3^, Craig Giragossian^3^, Aleksander S. Popel^1,2*^

*^1^Department of Biomedical Engineering, Johns Hopkins University School of Medicine, Baltimore, MD, USA*

*^2^Department of Oncology and Sidney Kimmel Comprehensive Cancer Center, Johns Hopkins University, Baltimore, MD, USA*

*^3^Biotherapeutics Discovery Research, Boehringer Ingelheim Pharmaceuticals, Inc, Ridgefield, CT, USA*

^*^Corresponding author email: hma24@jhmi.edu

Supplementary Information

**Bivalent TCE dynamics**

CEA and CD3 density on cancer, Teff and Treg cells were determined by equation 1-3. Here the C_CEA_total is the total number of CEA per cancer cell, Teff_CD3_total and Treg_CD3_total are the total number of CD3 per Teff or Treg cell, respectively. The total number of CD3 and CEA per cell was taken from literature data.

D_CEA_ = $\frac{C\_CEA\_total}{SA\_Ccell}$ (1)

D_TeffCD3_ = $\frac{Teff\_CD3\_total}{SA\_Tcell}$ (2)

D_TregCD3_ = $\frac{Treg\_CD3\_total}{SA\_Tcell}$ (3)

Binding of TCE to cancer cells was determined from D_CEA_ and CEA binding affinity including *k_on, CEA_TCE_, k_off, CEA_TCE_* and intrinsic cross-arm binding efficiency λ (Eqs. 4 and 5). D_syn_ denotes the immunological synapse gap distance and N is Avogadro constant. Similarly, binding of TCE and T cells (Teff or Treg) was calculated based on D_TeffCD3_ or D_TregCD3_ and CD3 binding affinity including *k_on, CD3_TCE_, k_off, CD3_TCE_* (Eqs. 6-1,6-2 and 7-1,7-2). In the tumor compartment, TCE can bind simultaneously to cancer cell to form CEA_TCE, CEACEA_TCE and to T cells to form CD3_TCE. These intermediate products will finally find the target on the other cell to form active final product CEACEA_TCE_CD3 (Eqs. 8-1,8-2). The formation of CEACEA_TCE_CD3 in the immunological synapse will cause enhanced cancel killing by Teff since more Teff cells are activated by TCE in an MHC-independent manner. Treg will also be activated by TCEs to exhibit an immunoregulatory function, which is achieved by suppressing the activity of Teff cells in this model.

$\frac{d(CEA\_TCE)}{dt}={2*k}_{on,CEA\_TCE}\cdot D_{CEA}\cdot\frac{TCE}{f_{tum}}-k_{off,CEA\_TCE}\cdot D_{CEA\_TCE}-$ $\frac{\lambda}{D_{syn}*N}*k_{on,CEA\_TCE}\cdot D_{CEA\_TCE}\cdot D_{CEA}+{2*k}_{off,CEA\_TCE}\cdot D_{CEACEA\_TCE} - k_{on,CD3\_TCE}\cdot D_{CEA\_TCE}\cdot D_{TeffCD3}+k_{off,CD3\_TCE}\cdot D_{CEA\_TCE\_TeffCD3} - k_{on,CD3\_TCE}\cdot D_{CEA\_TCE}\cdot D_{TregCD3} +k_{off,CD3\_TCE}\cdot D_{CEA\_TCE\_TregCD3}$ (4)

$\frac{d(CEACEA\_TCE)}{dt}=\frac{\lambda}{D_{syn}*N}*k_{on,CEA\_TCE}\cdot D_{CEA\_TCE}\cdot D_{CEA}-{2*k}_{off,CEA\_TCE}\cdot D_{CEACEA\_TCE} - k_{on,CD3\_TCE}\cdot D_{CEACEA\_TCE}\cdot D_{TeffCD3} +k_{off,CD3\_TCE}\cdot D_{CEACEA\_TCE\_TeffCD3} - k_{on,CD3\_TCE}\cdot D_{CEACEA\_TCE}\cdot D_{TregCD3}+k_{off,CD3\_TCE}\cdot D_{CEACEA\_TCE\_TregCD3}$ (5)

$\frac{d(TeffCD3\_TCE)}{dt}=k_{on,CD3\_TCE}\cdot D_{TeffCD3}\cdot\frac{TCE}{f_{tum}}-k_{off,CD3\_TCE}\cdot D_{TeffCD3\_TCE}$ - ${2*k}_{on,CEA\_TCE}\cdot D_{TeffCD3\_TCE}\cdot D_{CEA}+k_{off,CEA\_TCE}\cdot D_{CEA\_TCE\_TeffCD3}$ (6-1)

$\frac{d(TregCD3\_TCE)}{dt}=k_{on,CD3\_TCE}\cdot D_{TregCD3}\cdot\frac{TCE}{f_{tum}}-k_{off,CD3\_TCE}\cdot D_{TregCD3\_TCE}$ - ${2*k}_{on,CEA\_TCE}\cdot D_{TregCD3\_TCE}\cdot D_{CEA}+k_{off,CEA\_TCE}\cdot D_{CEA\_TCE\_TregCD3}$ (6-2)

$\frac{d(CEA\_TCE\_TeffCD3)}{dt}=k_{on,CD3\_TCE}\cdot D_{CEA\_TCE}\cdot D_{TeffCD3}-k_{off,CD3\_TCE}\cdot D_{CEA\_TCE\_TeffCD3}$ + ${2*k}_{on,CEA\_TCE}\cdot D_{TeffCD3\_TCE}\cdot D_{CEA}-k_{off,CEA\_TCE}\cdot D_{CEA\_TCE\_TeffCD3} {- \frac{\lambda}{D_{syn}*N}*k}_{on,CEA\_TCE}\cdot D_{CEA\_TCE\_TeffCD3}\cdot D_{CEA}+{2*k}_{off,CEA\_TCE}\cdot D_{CEACEA\_TCE\_TeffCD3} ($7-1)

$\frac{d(CEA\_TCE\_TregCD3)}{dt}=k_{on,CD3\_TCE}\cdot D_{CEA\_TCE}\cdot D_{TregCD3}-k_{off,CD3\_TCE}\cdot D_{CEA\_TCE\_TregCD3}$ + ${2*k}_{on,CEA\_TCE}\cdot D_{TregCD3\_TCE}\cdot D_{CEA}-k_{off,CEA\_TCE}\cdot D_{CEA\_TCE\_TregCD3} -{\frac{\lambda}{D_{syn}*N}*k}_{on,CEA\_TCE}\cdot D_{CEA\_TCE\_TregCD3}\cdot D_{CEA}+{2*k}_{off,CEA\_TCE}\cdot D_{CEACEA\_TCE\_TregCD3} ($7-2)

$\frac{d(CEACEA\_TCE\_TeffCD3)}{dt}={\frac{\lambda}{D_{syn}*N}*k}_{on,CEA\_TCE}\cdot D_{CEA\_TCE\_TeffCD3}\cdot D_{CEA}-{2*k}_{off,CEA\_TCE}\cdot D_{CEACEA\_TCE\_TeffCD3}$ + $k_{on,CD3\_TCE}\cdot D_{CEACEA\_TCE}\cdot D_{TeffCD3}-k_{off,CD3\_TCE}\cdot D_{CEACEA\_TCE\_TeffCD3}$ (8-1)

$\frac{d(CEACEA\_TCE\_TregCD3)}{dt}={\frac{\lambda}{D_{syn}*N}*k}_{on,CEA\_TCE}\cdot D_{CEA\_TCE\_TregCD3}\cdot D_{CEA}-{2*k}_{off,CEA\_TCE}\cdot D_{CEACEA\_TCE\_TregCD3}$ + $k_{on,CD3\_TCE}\cdot D_{CEACEA\_TCE}\cdot D_{TregCD3}-k_{off,CD3\_TCE}\cdot D_{CEACEA\_TCE\_TregCD3}$ (8-2)

Here *f_tum_* is the porosity in the tumor. The number of bound CEACEA_TCE_CD3 was translated to cancer cell killing rate by Teff cells using a Hill equation and the number of CEACEA_TCE_TregCD3 was translated to Teff exhaustion by Treg using another Hill equation. The baseline parameters used in this module were determined by fitting the model results to published *in vitro* experimental outcomes explained in the main text. The immune checkpoint blockade dynamics elaborated by Jafarnejad et al. (1). was also considered as part of the Teff cell killing rate (TKR) though it is close to 0 since no immune checkpoint inhibitors were administrated during this study. Teff’s death rate (TDR) was calculated from basal exhaustion and additional death rate caused by TCE-activated Treg.

*H_CEA_C1_T1*$=\text{ }\frac{{CEACEA\_TCE\_TeffCD3}^{3}}{{CEACEA\_TCE\_TeffCD3}^{3}+\text{ }K_{CEACEA\_TCE\_TeffCD3}^{3}}$ (9)

$TKR =\text{ }k_{C,death,{TCET}_{eff}}\frac{C \cdot\text{ }T_{eff}}{C +\text{ }T_{tot}}* H\_CEA\_C1\_T1$ + $k_{C,death,T_{eff}}\frac{C \cdot\text{ }T_{eff}}{C +\text{ }T_{tot}}\left( 1-\frac{{PD1\_PDLX}^{2}}{{PD1\_PDLX}^{2}+\text{ }K_{PD1\_PDLX}^{2}} \right)$

(10)

*H_CEA_C1_T0*$=\text{ }\frac{{CEACEA\_TCE\_TregCD3}^{3}}{{CEACEA\_TCE\_TregCD3}^{3}+\text{ }K_{CEACEA\_TCE\_TregCD3}^{3}}$ (11)

$TDR =\text{ }k_{Teff,death}\frac{Treg \cdot\text{ }T_{eff}}{C +\text{ }T_{tot}}*(1+H\_CEA\_C1\_T0)$ (12)

Here $k_{C,death,T_{eff}}$ is basal cancer killing rate by Teff and $\text{k}_{\text{C,death,}{\text{TCE}\text{T}}_{\text{eff}}}$ is additional cancer killing rate by Teff activated by TCE, *C* is the total number of cancer cells in the tumor compartment, $\text{T}_{\text{eff}}$ is total number of Teff in the tumor and $\text{T}_{\text{tot}}$ is total T cells in the tumor, $CEACEA\_TCE\_TeffCD3$is the total number of engaged CEA CD3 molecules bridged by TCE in the synapse, and $\text{K}_{\text{CEACEA\_TCE\_TeffCD3}}$ is sensitivity of *TKR* to $CEACEA\_TCE\_TeffCD3$. Formation of $CEACEA\_TCE\_TeffCD3$ will increase *TKR* due to the Hill equation (H_CEA_C1_T1). k_(C,death,T_eff ) is basal Teff death rate by Treg and $\text{T}_{\text{reg}}$ is total number of Treg in the tumor.

Supplementary Figures

**Pharmacokinetics**

Pharmacokinetic of cibisatamab was modelled following the same physiologically-based pharmacokinetic model as described by Jafarnejad et al. (1). The plasma concentration of cibisatamab in our model was fitted to standard pharmacokinetic two-compartment model (Fig. S1). PK parameters were fitted to the data reported and the simulated plasma concentration of cibisatamab together with the clinical measurements at dose levels of 80, 160, 200, 300, 400 mg in the central compartment (2).

*TCE_P_*, *TCE_C_*, *TCE_LN_*, *TCE_T_* indicate TCE concentration in peripheral, central, TDLN and tumor compartment, respectively.

$V_{C}\frac{d{TCE}_{C}}{dt}=q_{P}\left( {TCE}_{P}-{TCE}_{C} \right)+q_{LN}\left( {TCE}_{LN}-{TCE}_{C} \right)+q_{T}\left( {TCE}_{T}-{TCE}_{C} \right)+q_{LD}{TCE}_{LN}-CL*{TCE}_{C} ($10)

$V_{P}\frac{d{TCE}_{P}}{dt}=q_{P}\left( {TCE}_{C}-{TCE}_{P} \right) ($11)

$V_{T}\frac{dTCE}{dt}=q_{T}\left( {TCE}_{C}-{TCE}_{T} \right)-q_{LD}{TCE}_{T} ($12)

$V_{LN}\frac{d{TCE}_{LN}}{dt}=q_{LN}\left( {TCE}_{C}-{TCE}_{LN} \right)+q_{LD}{TCE}_{T}-q_{LD}{TCE}_{LN} ($13)


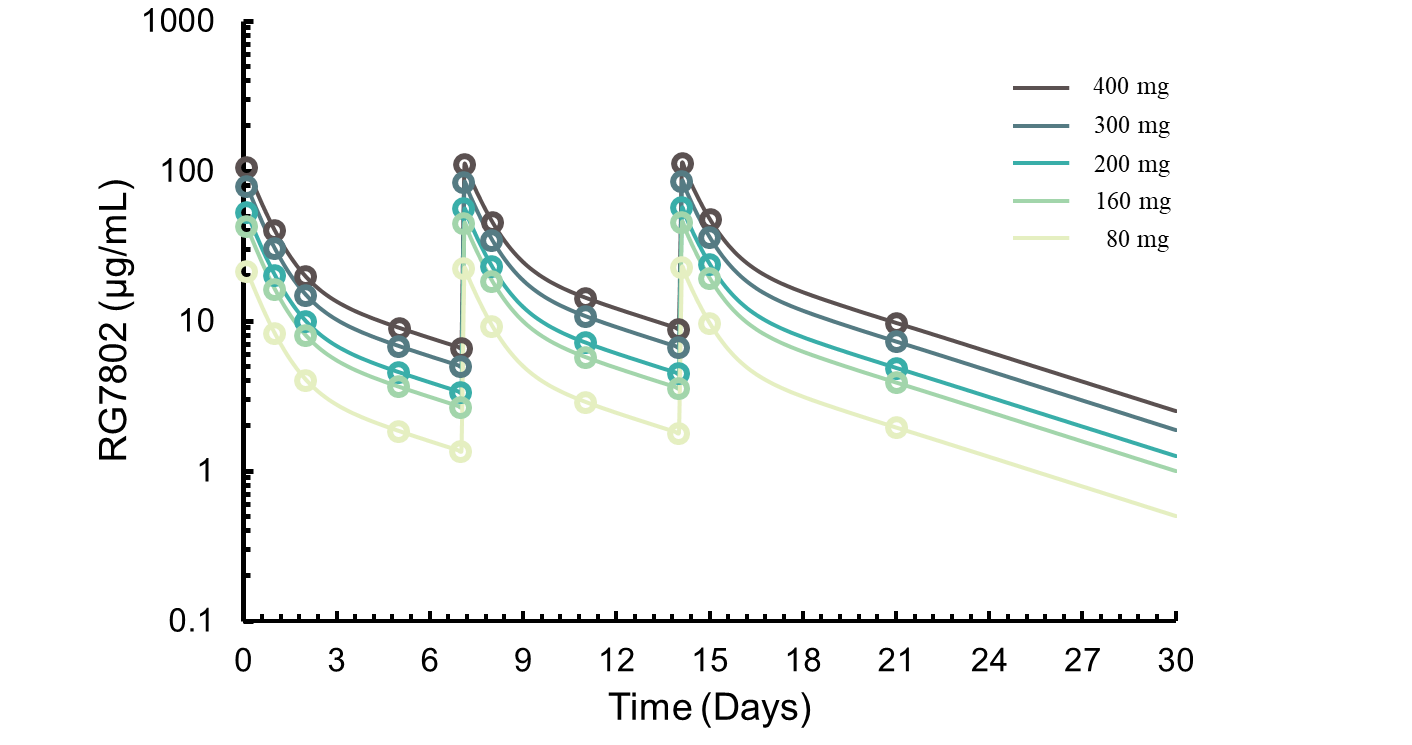


Fig. S1 Simulated (solid lines) and measured (dots) cibisatamab plasma concentration


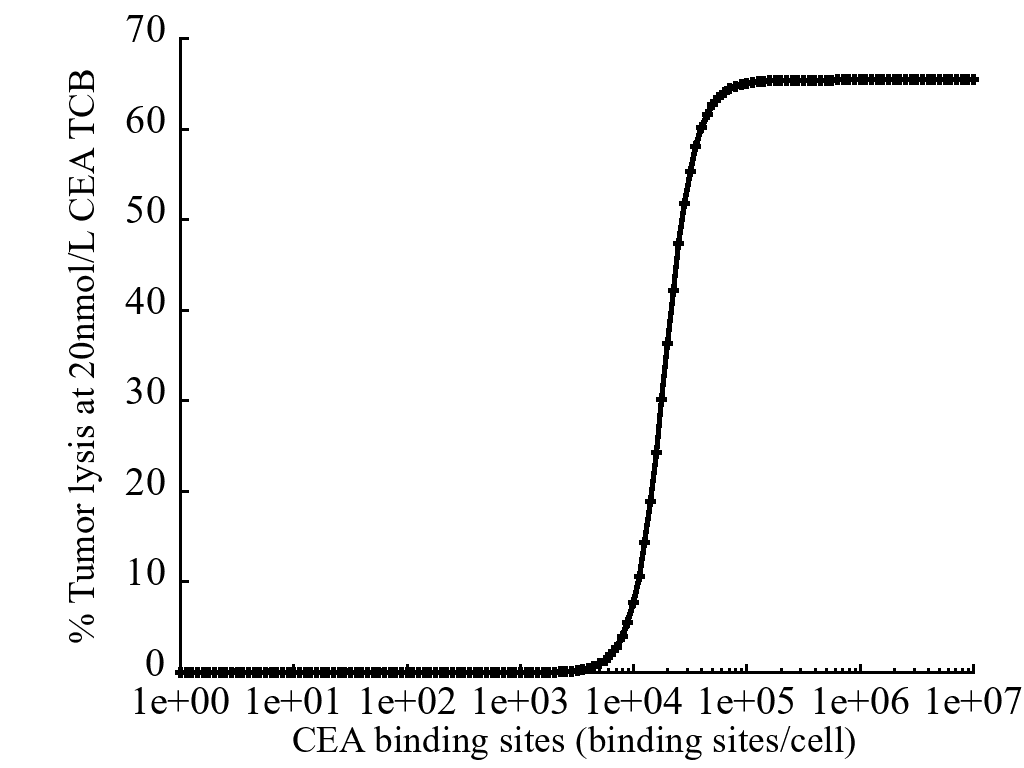


Fig. S2 The percentage of tumor cell lysis mediated by 20 nmol/L TCE


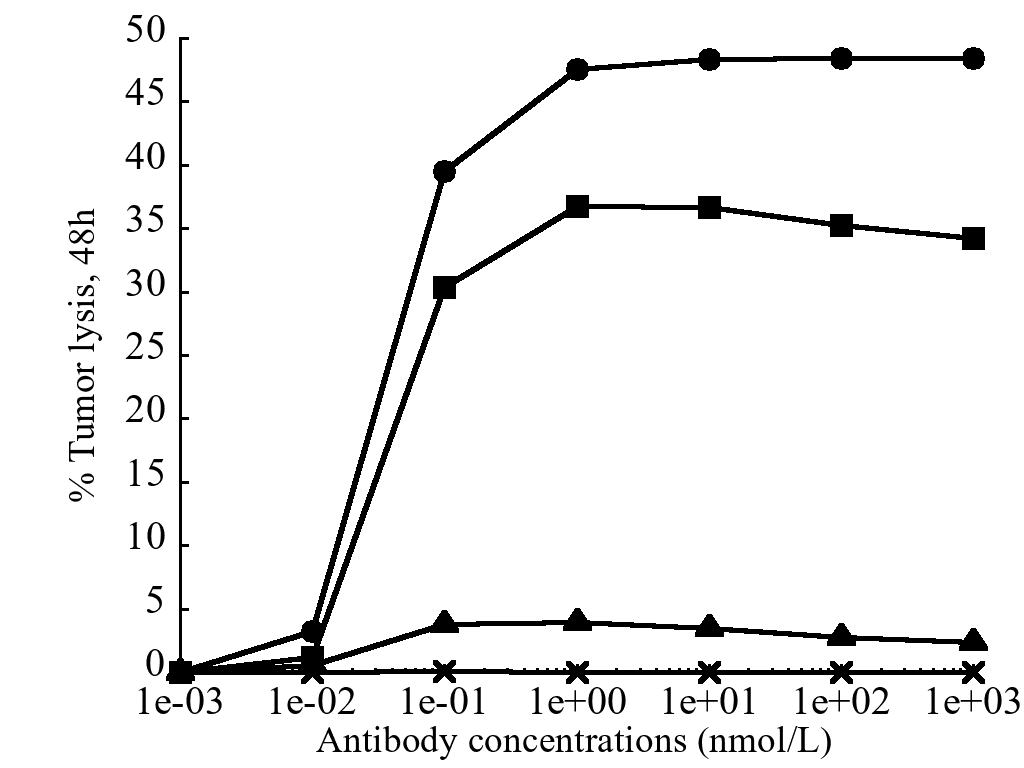


Fig. S3 Analysis of tumor cell lysis 48 hours after incubation with TCE and

human Teff cells.


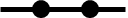
MKN45
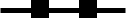
 LS174T
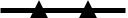
 HT-29
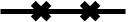
 CCD-841

B

**
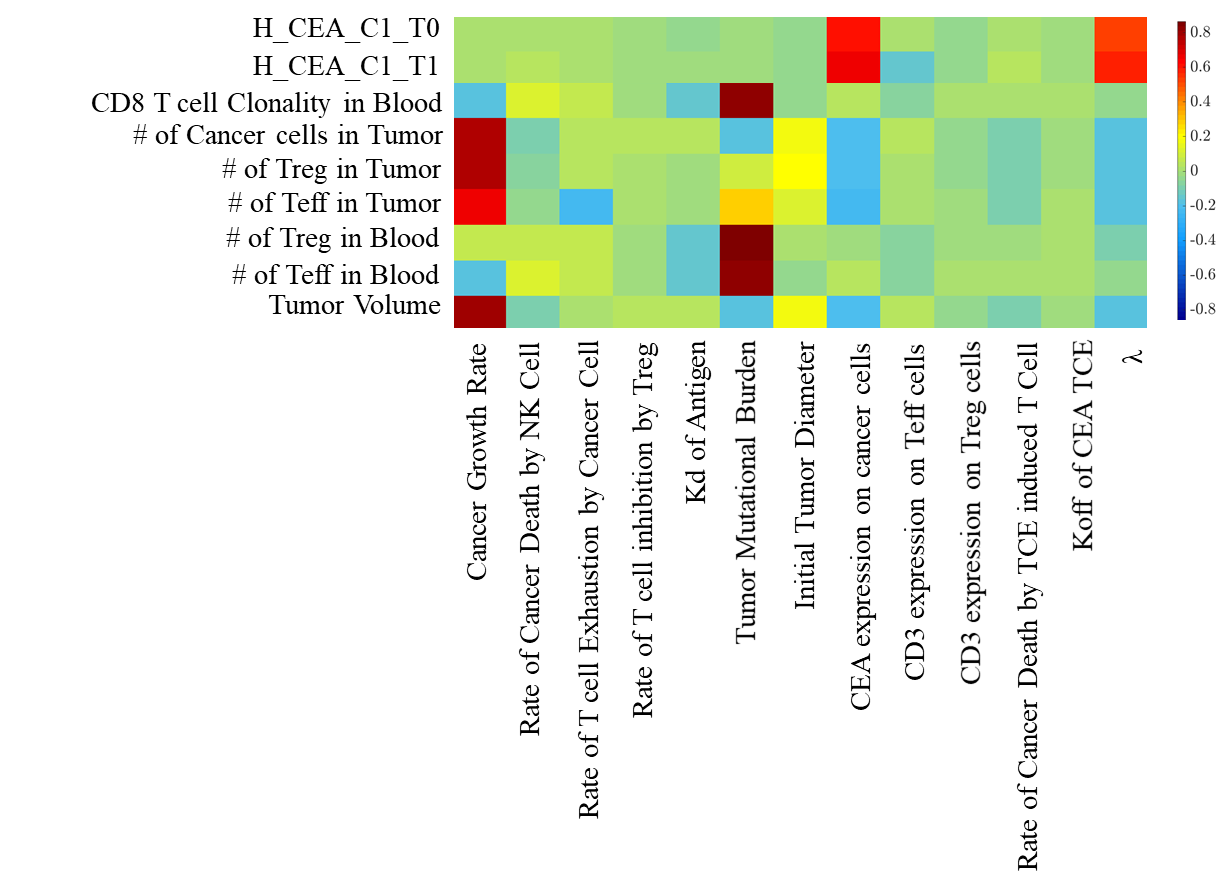
**

A

**
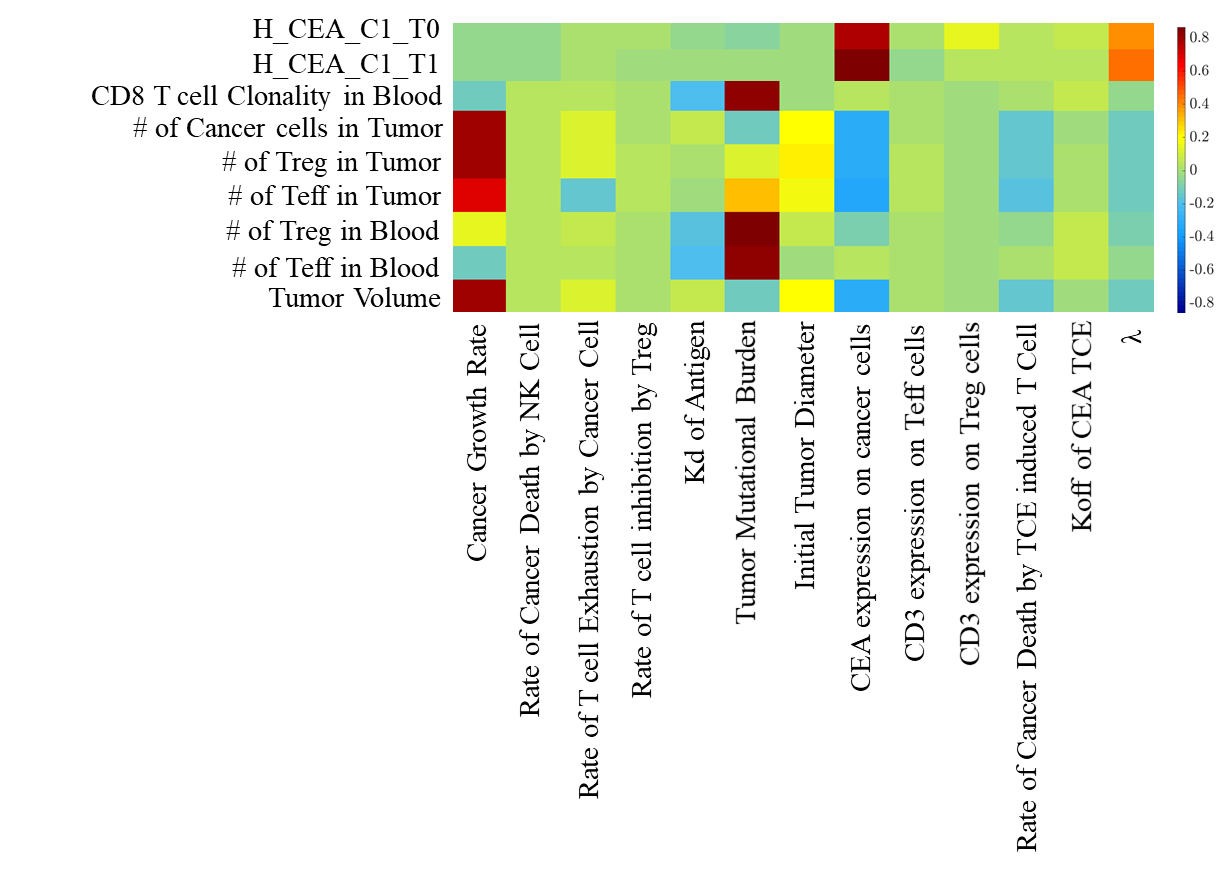
**

B

Figure S4. The partial rank correlation coefficient, PRCC, for individual parameters A. CEA expression = 10000 – 300000, B. CEA expression = 300000 – 1000000

Supplementary Table

Table. S1 Abbreviations

| **Abbreviation** | **Definition** |
| --- | --- |
| biTTC | bivalent TCEs Ternary Complex |
| CEA | Carcinoembryonic Antigen |
| CEA-TCB | Carcinoembryonic Antigen T-Cell Bispecific Antibody |
| CR | Complete Response |
| CTLA-4 | cytotoxic T-lymphocyte-associated protein 4 |
| EpCAM | Epithelial cell adhesion molecule |
| Fc | Fragment crystallizable |
| LHS | Latin hypercube sampling |
| mAb | Monoclonal antibody |
| mCRC | metastatic Colorectal Cancer |
| MHC | Major Histocompatibility Complex |
| moTTC | monovalent TCEs Ternary Complex |
| MSS | Microsatellite Stable |
| NSCLC | Non-small-cell Lung Carcinoma |
| PBMC | Peripheral Blood Mononuclear Cell |
| PD | Progressive Disease |
| PD-1 | Programmed cell death protein 1 |
| PD-L1 | Programmed death-ligand 1 |
| pMMR | Mismatch Repair proficient |
| PR | Partial Response |
| PRCC | Partial Rank Correlation Coefficient |
| PSA | Parameter Sensitivity Analysis |
| QSP | Quantitative Systems Pharmacology |
| SBML | Systems Biology Markup Language |
| scFv | Single-chain variable fragment |
| SD | Stable Disease |
| TCB | T Cell Bispecific |
| TCE | T cell engager |
| TDLN | Tumor-draining Lymph Nodes |
| Teff | effector T cells |
| TMB | Tumor Mutational Burden |
| Treg | regulatory T cells |
| TTC | TCE-induced Ternary Complex |

Table. S2 Model Variables and Terms Used in Equations

| Variable | Definition | Unit | Value |
| --- | --- | --- | --- |
| C_CEA_total | Total CEA on tumor cell | sites/cell | 10000 |
| Teff_CD3_total | Total CD3 on Teff cell | sites/cell | 61000 |
| Treg_CD3_total | Total CD3 on Treg cell | sites/cell | 61000 |
| Kd_CEATCE | Binding affinity of CEA/TCE | nM | 130 |
| Kon_CEATCE | On rate constant for CEA/TCE binding | 1/(M*s) | 1000 |
| Koff_CEATCE | Off rate constant for CEA/TCE binding | 1/s | 0.00013 |
| Kd_CD3TCE | Binding affinity of CD3/TCE | nM | 75 |
| Kon_CD3TCE | On rate constant for CD3/TCE binding | 1/(M*s) | 10000 |
| Koff_CD3TCE | Off rate constant for CD3/TCE binding | 1/s | 0.00075 |
| λ | Intrinsic antibody cross-arm binding efficiency | dimensionless | 1000 |
| n_CEATCE | Hill coefficient for CEATCE | Dimensionless | 3 |
| CEA_TCE | Dimer of CEA_TCE in tumor comp | Molecule |  |
| CEACEA_TCE | Dimer of CEA_TCE_CEA in tumor comp | Molecule |  |
| TeffCD3_TCE | Dimer of TeffCD3_TCE in tumor comp | Molecule |  |
| TregCD3_TCE | Dimer of TregCD3_TCE in tumor comp | Molecule |  |
| CEA_TCE_CD3 | Dimer of CD3_TCE_CEA in tumor comp | Molecule |  |
| CEA_TCE_TeffCD3 | moTTC of CEA_TCE_TeffCD3 in tumor comp | Molecule |  |
| CEA_TCE_TregCD3 | moTTC of CEA_TCE_TregCD3 in tumor comp | Molecule |  |
| CEACEA_TCE_TeffCD3 | Trimer of CEACEA_TCE_TeffCD3 in tumor comp | Molecule |  |
| CEACEA_TCE_TregCD3 | Trimer of CEACEA_TCE_TregCD3 in tumor comp | Molecule |  |
| V_LN.T1 | Total T cells in the LN comp | Cell |  |
| V_T.T1 | Total T cells in the Tumor comp | Cell |  |
| V_C.T1 | Total T cells in the central comp | Cell |  |
| V_P.T1 | Total T cells in the Peripheral comp | Cell |  |
| V_LN.TCE | Concentration of TCE in LN comp | M |  |
| V_T.TCE | Concentration of TCE in tumor comp | M |  |
| V_C.TCE | Concentration of TCE in central comp | M |  |
| V_P.TCE | Concentration of TCE in Peripheral comp | M |  |

Table. S3 Parameter Values and Ranges Used in the Sensitivity Analysis

| Parameter | Baseline Value | Sensitivity test range | Unit |
| --- | --- | --- | --- |
| Tumor Growth Rate | 0.005 | 0-0.05 | 1/day |
| Rate of cancer death by NK cell | 0.00001 | 0.00001-0.001 | 1/day |
| Rate of T cell exhaustion by cancer cell | 0.1 | 0.05-0.5 | 1/day |
| Rate of cancer death by T cell | 3 | 1-8 | 1/day |
| Rate of Teff inhibition by Treg | 1 | 0.1-1 | 1/day |
| K_D_ of Ag-MHC | 4.0E-08 | 4E-10 - 4E-6 | M |
| Number of Ag Clones (TMB) | 50 | 0 - 5E4 | dimensionless |
| Initial Tumor Diameter | 3 | 0.5-5 | cm |
| CEA expression in cancer cell | 20000 | 1000-300000 | Molecule |
| CD3 expression in cancer cell | 61000 | 30000-90000 | molecule |
| CD3 expression in cancer cell | 61000 | 30000-90000 | molecule |
| Rate of Tumor Death by TCE activated Teff | 1 | 1-10 | molecule |
| Rate of TCE activated Treg Inhibition of Teff | 2 | 0.1-10 | 1/day |
| koff of CEA TCE | 0.00013 | 0.000001-0.001 | 1/s |
| koff of CD3 TCE | 0.00075 | 0.00001-0.01 | 1/s |
| λ | 1000 | 0.001-100000 | dimensionless |

**References**

1. Jafarnejad M, Gong C, Gabrielson E, Bartelink IH, Vicini P, Wang B, et al. A Computational Model of Neoadjuvant PD-1 Inhibition in Non-Small Cell Lung Cancer. Aaps Journal. 2019;21(5):79.
2. Ignacio, M. et al. Pharmacokinetics (PK) and pharmacodynamics (PD) of a novel carcinoembryonic antigen (CEA) T-cell bispecific antibody (CEA CD3 TCB) for the treatment of CEA-expressing solid tumors. J. Clin. Oncol. 2017; 35: 2549-2549.
